# HDAC1-3 inhibitor MS-275 enhances *IL10* expression in RAW264.7 macrophages and reduces cigarette smoke-induced airway inflammation in mice

**DOI:** 10.1038/srep45047

**Published:** 2017-03-27

**Authors:** Niek G. J. Leus, Thea van den Bosch, Petra E. van der Wouden, Kim Krist, Maria E. Ourailidou, Nikolaos Eleftheriadis, Loes E. M. Kistemaker, Sophie Bos, Rutger A. F. Gjaltema, Solomon A. Mekonnen, Rainer Bischoff, Reinoud Gosens, Hidde J. Haisma, Frank J. Dekker

**Affiliations:** 1 Department of Chemical and Pharmaceutical Biology, Groningen Research Institute of Pharmacy (GRIP), University of Groningen, Antonius Deusinglaan 1, 9713 AV, Groningen, The Netherlands; 2 Department of Molecular Pharmacology, Groningen Research Institute of Pharmacy (GRIP), University of Groningen, Antonius Deusinglaan 1, 9713 AV, Groningen, The Netherlands; 3 Department of Pathology & Medical Biology, University Medical Center Groningen (UMCG), University of Groningen, Hanzeplein 1, 9713 GZ, Groningen, The Netherlands; 4 Department of Analytical Biochemistry, Groningen Research Institute of Pharmacy (GRIP), University of Groningen, Antonius Deusinglaan 1, 9713 AV, Groningen, The Netherlands

## Abstract

Chronic obstructive pulmonary disease (COPD) constitutes a major health burden. Studying underlying molecular mechanisms could lead to new therapeutic targets. Macrophages are orchestrators of COPD, by releasing pro-inflammatory cytokines. This process relies on transcription factors such as NF-κB, among others. NF-κB is regulated by lysine acetylation; a post-translational modification installed by histone acetyltransferases and removed by histone deacetylases (HDACs). We hypothesized that small molecule HDAC inhibitors (HDACi) targeting class I HDACs members that can regulate NF-κB could attenuate inflammatory responses in COPD via modulation of the NF-κB signaling output. MS-275 is an isoform-selective inhibitor of HDAC1-3. In precision-cut lung slices and RAW264.7 macrophages, MS-275 upregulated the expression of both pro- and anti-inflammatory genes, implying mixed effects. Interestingly, anti-inflammatory *IL10* expression was upregulated in these model systems. In the macrophages, this was associated with increased NF-κB activity, acetylation, nuclear translocation, and binding to the *IL10* promoter. Importantly, in an *in vivo* model of cigarette smoke-exposed C57Bl/6 mice, MS-275 robustly attenuated inflammatory expression of KC and neutrophil influx in the lungs. This study highlights for the first time the potential of isoform-selective HDACi for the treatment of inflammatory lung diseases like COPD.

Chronic obstructive pulmonary disease (COPD) is associated with chronic inflammatory responses. For most COPD patients the currently available therapy is not effective due to reduced responsiveness to corticosteroids^1^,^2^,^3^. COPD constitutes a major health burden, and underlying molecular mechanisms that can be targeted to devise new therapeutic strategies need to be studied.

Cigarette smoke is a major etiological factor in COPD^4^, and in susceptible individuals results in severe lung inflammation characterized by influx of inflammatory cells and secretion of cytokines^5^. Lungs of COPD patients have increased numbers of macrophages, which may be explained by increased recruitment of monocytes in response to chemokines produced in the lungs. Macrophages play a pivotal role in COPD as they secrete both pro-inflammatory cytokines such as TNF-α, IL-1 and IL-8, as well as the anti-inflammatory cytokine IL-10^6^,^7^. This suggests that underlying regulatory processes in macrophages are important in balancing between expression of pro- or anti-inflammatory genes.

The NF-κB transcription factor regulates inflammatory gene transcription, and is involved in the expression of important pro-inflammatory cytokines in macrophages in COPD^6^,^7^. Interestingly, a role for the NF-κB pathway has been implicated in COPD, as reviewed elsewhere^8^. Increased nuclear localization of p65 was observed in sputum macrophages during exacerbations of COPD^9^, and in bronchial biopsies of COPD patients^10^. Research has focused on modulating NF-κB activity as a therapeutic strategy for COPD treatment^8^, highlighting NF-κB as a potential therapeutic target.

Lysine acetylation is an important post-translational modification found on numerous cellular proteins, including both histone and non-histone proteins. Steady-state acetylation levels result from the balance between the opposing activities of enzymes called histone acetyltransferases (HATs) and histone deacetylases (HDACs)^11^. Lysine acetylations of histones are part of the epigenetic code for regulation of gene transcription^12^. For NF-κB, the p65 subunit in particular is subject to dynamic lysine acetylations that affect transcriptional capacity, DNA binding and duration of action^13^. Acetylations of the NF-κB p65 subunit on lysines 218, 221 and 310 increase transcriptional activity^14^, whereas acetylations on lysines 122 and 123 decrease transcriptional activity^15^. HDAC-NF-κB interactions are important in NF-κB regulation but have not been fully understood. Studies indicate that some class I HDAC members could be important. It has been demonstrated that HDAC1 knockdown reduced LPS-induced gene expression of *iNOS* and *IL6* in bone marrow-derived macrophages^16^. In contrast it has been demonstrated that HDAC1 negatively regulated NF-κB-mediated gene transcription through association with p65, whereas HDAC2 does not interact with NF-κB directly but regulates NF-κB activity via its interaction with HDAC1^17^. HDAC3 has been reported to deacetylate specific lysines of NF-κB p65^18^. It was shown that HDAC3 is required for IL-1-induced gene expression, where the positive regulatory role of HDAC3 involves binding to the NF-κB p65 subunit and deacetylation of various inhibitory lysine acetylations^19^. Also, HDAC3-deficient macrophages are unable to activate a subset of the LPS-induced inflammatory gene expression, and HDAC3 ensures that NF-κB is kept in a primarily deacetylated active state^20^. Altogether, this suggests that selective inhibition of HDAC1-3 could alter the balance between pro- and anti-inflammatory gene expression via regulation of NF-κB acetylation, thereby suppressing inflammation in COPD.

Small molecule HDAC inhibitors (HDACi) are used in the treatment of haematological cancers^21^. Among zinc-dependent HDACs, these FDA approved HDACi are non-selective; but a number of more selective HDACi are in clinical trials^21^. Interestingly, studies indicate that HDACi can suppress inflammatory responses at much lower concentrations than those used for cancer treatment^22^. MS-275 is a HDACi currently in clinical trials, which is selective for HDAC1, 2 and 3^21^. Effects of MS-275 on NF-κB have been reported before, as well as effects in various disease models characterized by inflammation. MS-275 inhibited LPS-induced NF-κB p65 nuclear accumulation in human fibroblastic cells, gave rise to less acetylated NF-κB p65 in the nucleus, and reduced the secretion of pro-inflammatory cytokines IL-6 and IL-18^23^. In sciatic nerves and lymph nodes of experimental autoimmune neuritis rats, MS-275 downregulated pro-inflammatory gene expression^24^. In a rat experimental autoimmune prostatitis model, MS-275 reduced infiltration of inflammatory cells into the prostate and reduced mRNA levels of inflammatory genes *TNFα, IFNγ, IL17*, and *iNOS*^25^. Together, these lines of evidence indicate that MS-275 has potential to modulate NF-κB, and to attenuate inflammatory responses. Thus, we hypothesize that small molecules interfering with the activity of specific HDAC iso-enzymes, such as MS-275, have the potential to suppress inflammation in COPD model systems. We anticipate that the precise effects of HDACi depend on their selectivity for the HDACs that target specific NF-κB acetylation sites, suggesting that selective inhibition of HDACs might result in selective effects on inflammatory responses.

Here, we studied MS-275 in model systems for inflammatory responses. Effects of MS-275 on pro- and anti-inflammatory gene expression were investigated in precision-cut lung slices (PCLS), and RAW264.7 macrophages, that were stimulated with LPS/IFNγ. Increased *IL10* expression was observed in PCLS as well as RAW264.7 macrophages upon MS-275 treatment. The underlying mechanism was studied in the macrophages, and was connected to increased NF-κB p65 transcriptional activity, acetylation, nuclear localization and promoter binding. Finally, MS-275 treatment was studied in cigarette smoke-induced neutrophilic airway inflammation in C57Bl/6 mice; a relevant and commonly used COPD model^26^,^27^. MS-275 was nebulized, allowing for the advantage of local administration by inhalation. Importantly, in this model, inflammatory gene expression levels and inflammatory cell recruitment were reduced in the lungs upon MS-275 treatment. To our knowledge, this study is the first to demonstrate that MS-275 robustly attenuates cigarette smoke-induced neutrophilic airway inflammation in C57Bl/6 mice.

## Results

### MS-275 affects both pro- and anti-inflammatory gene expression in mouse PCLS and is particularly effective in affecting inflammatory responses in macrophages

The selectivity of MS-275 as previously described^28^,^29^, was confirmed by determining IC_50_ values for recombinant human HDAC1-3, HDAC6 and HDAC8. MS-275 indeed selectively inhibits HDAC1-3 at low micromolar concentrations, while HDAC6 and HDAC8 were not inhibited at such concentrations (Table 1). For comparison the well-studied pan-HDACi SAHA was included, which was indeed non-selective among these HDACs.

**Figure 1:**
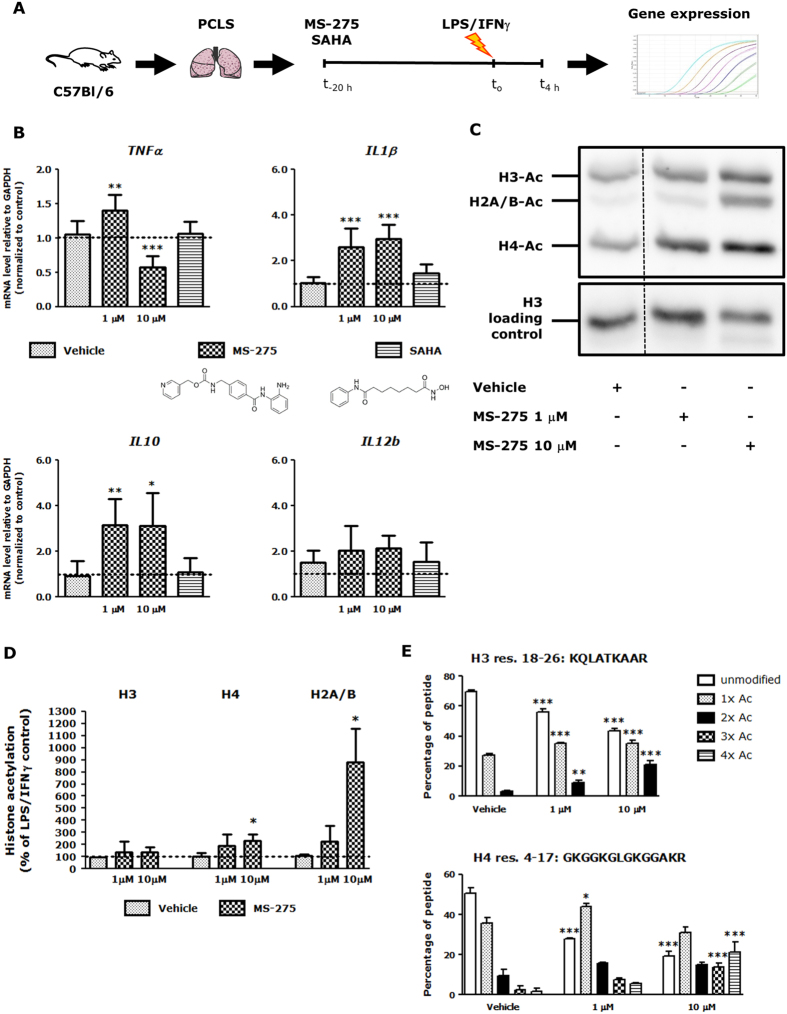
MS-275 affects inflammatory gene expression and histone acetylation in precision-cut lung slices. Schematic representation of the experimental setup and timeline **(A)**. Mouse precision-cut lung slices (PCLS) were subjected to MS-275 (1 μM or 10 μM) or SAHA (0.41 μM) for 20 h and stimulated with LPS/IFNγ the last 4 h of the experiment, and lysed. Gene expression was studied by RT-qPCR and expressed as fold change compared to control (LPS/IFNγ-treated) **(B**). Data are presented as mean ± SD of 5–11 independent experiments. *p < 0.05, **p < 0.01; ***p < 0.001 compared to vehicle (LPS/IFNγ and inhibitor solvent-treated). Under the same conditions, PCLS histones were extracted, resolved by SDS-PAGE, and effects of MS-275 on total histone acetylation were assessed by Western blot (dotted line indicates crop within the same blot; full length blot is presented in Figure S2) **(C)** and quantified by densitometry (**D)**. Data are presented as mean ± SD of 2–3 independent experiments. *p < 0.05 compared to vehicle (LPS/IFNγ and inhibitor solvent-treated). Histones H3 and H4 were also excised from the gel and subjected to LC-MS/MS analysis **(E)**. MS-275 increased acetylation on one peptide from histone H3 (res. peptide 18–26: KQLATKAAR) and one peptide from histone H4 (res. peptide 4–17: GKGGKGLGKGGAKR). Other peptides were not affected. Data are presented as mean ± SD of 3 independent experiments. *p < 0.05; **p < 0.01; ***p < 0.001 compared to vehicle (LPS/IFNγ and inhibitor solvent-treated).

Mouse PCLS (Fig. 1A) stimulated with LPS/IFNγ were used as a model system for inflammatory lung diseases for our studies with MS-275. PCLS contain all cell types present *in vivo* and conserve the original architecture and matrix environment^30^. The viability of PCLS upon MS-275 and SAHA treatment was studied by measuring release of lactate dehydrogenase (LDH) into the culture medium. Both HDACi did not affect PCLS viability at concentrations ≤10 μM (Figure S1). Expression of pro-inflammatory genes *TNFα, IL1β* and *IL12b*, and the anti-inflammatory gene *IL10*, were examined in PCLS. MS-275 upregulated *IL10* expression by 3-fold in PCLS, which was accompanied by downregulation of *TNFα* at 10 μM (Fig. 1B). The expression of *IL1β* was also upregulated. SAHA did not affect gene expression in PCLS (Fig. 1B). This demonstrates that iso-enzyme selectivity of HDACi is important to their influence on gene expression in this model; and that MS-275 has a mixed pro- and anti-inflammatory effect on gene expression.

Effects of MS-275 on histone acetylation in LPS/IFNγ-stimulated PCLS were investigated. Global histone acetylation was studied by Western blot (Fig. 1C) and quantified by densitometry (Fig. 1D). MS-275 at 10 μM increased acetylation of histone H2A/B and H4 compared to vehicle-treated PCLS (Fig. 1D). No significant effect was observed on acetylation of histone H3. Acetylation of histone H3 and H4 was assessed in more detail using high-resolution mass spectrometry. A previously described approach was employed in which histones are fully acetylated using deuterated acetic anhydride ((CD_3_CO)_2_O) followed by tryptic digestion^31^ (histone H2A/B are not analyzed using this method). Using this method a relative quantification of acetylated versus non-acetylated lysines of histone H3 and H4 in PCLS was obtained. Compared to western blot this allows for a more precise allocation of the changes in acetylation to individual histone peptides, and could allow for monitoring smaller changes in acetylation which could be missed by western blot. MS-275 dose-dependently increased acetylation of one peptide from histone H3 (res. 18–26: KQLATKAAR) and one from histone H4 (res. 4–17: GKGGKGLGKGGAKR) (Fig. 1E), whereas other peptides were unaffected (data not shown). Taken together, MS-275 increases histone acetylation in LPS/IFNγ-stimulated PCLS.

**Figure 2:**
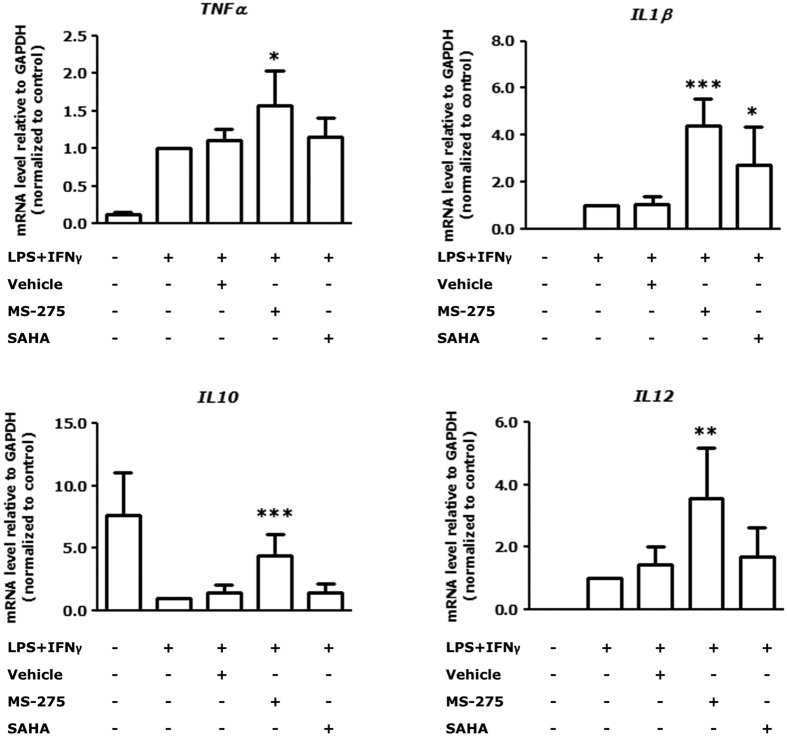
MS-275 enhances gene expression of pro- and anti-inflammatory genes in macrophages. RAW264.7 macrophages were incubated with HDACi MS-275 (1 μM) or SAHA (0.41 μM) for 20 h and stimulated with LPS/IFNγ the last 4 h of the experiment. Gene expression was studied by RT-qPCR and expressed as fold change compared to control (LPS/IFNγ-treated) group. Data are presented as mean ± SD of 4–8 independent experiments. *p < 0.05; **p < 0.01; ***p < 0.001 compared to vehicle (LPS/IFNγ and inhibitor solvent-treated).

We moved on to study molecular consequences of HDAC1-3 inhibition in a cell-based model system for inflammatory lung diseases using mouse RAW264.7 macrophages stimulated with LPS and IFNγ. With this we aimed to unravel whether macrophages play an important role in the effects observed for MS-275. First, cell viability upon MS-275 or SAHA treatment was tested. MTS assays demonstrated no significant decreases in cell viability (Figure S1; Table S1). In LPS/IFNγ-stimulated RAW264.7 macrophages, MS-275 treatment upregulated expression of *TNFα, IL1β, IL12b* and *IL10* (Fig. 2). Interestingly, SAHA, except for *IL1β*, did not affect gene expression in the macrophages (Fig. 2). The observed effects clearly demonstrate that HDAC1-3 inhibition is effective in affecting inflammatory responses in macrophages.

Taken together, the upregulation of both the anti-inflammatory gene *IL10* and the pro-inflammatory gene *IL1β* and downregulation of the pro-inflammatory gene *TNFα* in PCLS, and upregulation of *IL10, TNFα, IL1β, IL12b* in RAW264.7 macrophages, indicates potential mixed effects for HDAC1-3 inhibition by MS-275 in inflammation. The upregulation of *IL10* expression in the macrophages is particularly interesting because of its anti-inflammatory role. Therefore, the connection between MS-275 and *IL10* upregulation was further investigated in RAW264.7 macrophages, aiming to identify underlying molecular mechanisms.

### MS-275 increases LPS/IFNγ-induced NF-κB p65 transcriptional activity, acetylation, nuclear translocation, and binding at the *IL10* promoter in RAW264.7 macrophages

It has been described that LPS stimulation of macrophages activates NF-κB, which induces the expression of *IL10* along with *TNFα, IL1β*, and *IL12b*^32^. The observed mixed effects in RAW264.7 macrophages upon MS-275 treatment therefore fit a profile where NF-κB activity is further enhanced by MS-275. Therefore, we set out to characterize the contribution of this pathway to the changes in gene expression, particularly the increased *IL10* expression, induced by MS-275.

**Figure 3:**
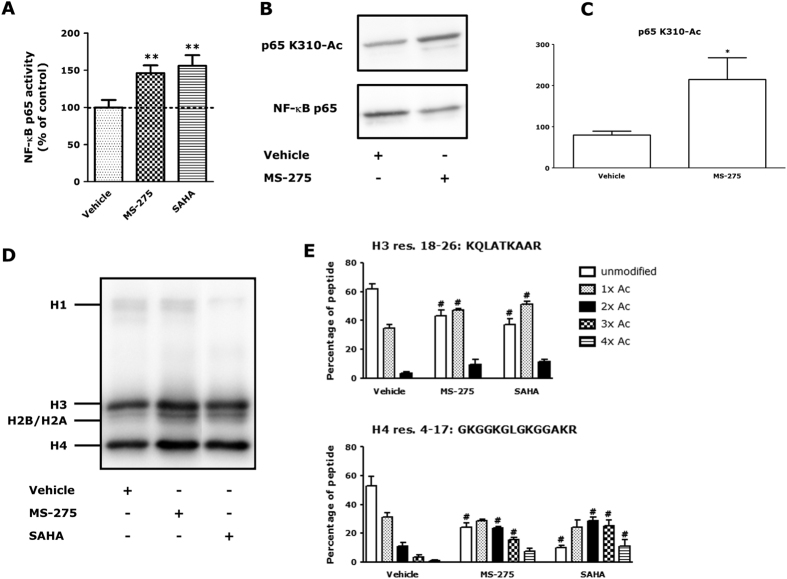
MS-275 enhances LPS/IFNγ-induced NF-κB p65 activity and increases NF-κB p65 acetylation and histone acetylation in RAW264.7 macrophages. RAW-Blue macrophages were subjected to 1 μM MS-275 or 0.41 μM SAHA for 20 h and stimulated with LPS/IFNγ the last 4 h of the experiment. Treatment of RAW-Blue cells with HDACi followed by LPS/IFNγ significantly increased NF-κB activity (**A**). Data are presented as mean ± SD of 3–4 independent experiments and control (LPS/IFNγ-treated) cells were set at 100%. **p < 0.01, compared to vehicle (LPS/IFNγ and inhibitor solvent-treated) cells. For detection of NF-κB p65 K310 acetylation, RAW264.7 macrophages were incubated with HDACi for 20 h, and lysed. Effects on NF-κB p65 K310 acetylation were studied by Western blot (the blots were cropped; full length blots are presented in Figure S3) (**B**) and quantified by densitometry compared to p65 as loading control (**C**). Data are presented as mean ± SEM expressed as fold change compared to vehicle (LPS/IFNγ and inhibitor solvent-treated) of 5–6 independent experiments. *p < 0.05, compared to vehicle (LPS/IFNγ and inhibitor solvent-treated). RAW264.7 histones were resolved by SDS-PAGE and blotted for detection of histone acetylation (blot was cropped; full length blot is shown in Figure S4) (**D**). Histones H3 and H4 were excised from the gel and subjected to LC-MS/MS analysis (**E**). MS-275 or SAHA increased acetylation on one peptide of histone H3 (res. 18–26: KQLATKAAR) and one of histone H4 (res. 4–17: GKGGKGLGKGGAKR). Other peptides were not affected. Data are presented as mean ± SD of 3–5 independent experiments. ^#^*p* < 0.001 compared to vehicle (inhibitor solvent-treated). No significant differences were observed between untreated and vehicle-treated cells (Figure S5).

Transcriptional activity of NF-κB p65 was studied using a reporter gene assay. NF-κB p65 promoter activity was significantly increased by 50% upon MS-275 treatment, which was also observed for SAHA (Fig. 3A). Changes in NF-κB p65 K310 acetylation status were studied by Western blot (Fig. 3B) and quantified by densitometry (Fig. 3C). MS-275 increased NF-κB p65 acetylation on K310 in LPS/IFNγ-stimulated RAW264.7 macrophages. This shows that MS-275 increases both transcriptional activity and acetylation status of NF-κB p65.

Histone acetylation was analyzed in RAW264.7 macrophages by Western blot. MS-275 increased acetylation of histone H3 and H4 compared to vehicle-treated cells (Fig. 3D). Acetylation of histone H3 and H4 was analyzed in more detail using mass spectrometry. Increased acetylation upon MS-275 and SAHA treatment was observed on one peptide from histone H3 (res. 18–26: KQLATKAAR) and one peptide from histone H4 (res. 4–17: GKGGKGLGKGGAKR) (Fig. 3E). Other peptides did not show changes in acetylation upon MS-275 treatment (data not shown). These observations are in line with the data for histone acetylation in PCLS. Altogether, these results demonstrated that both MS-275 and SAHA increased acetylation of one peptide on histone H3 and H4, whereas, interestingly, only MS-275, and not SAHA, affected NF-κB p65 acetylation.

**Figure 4:**
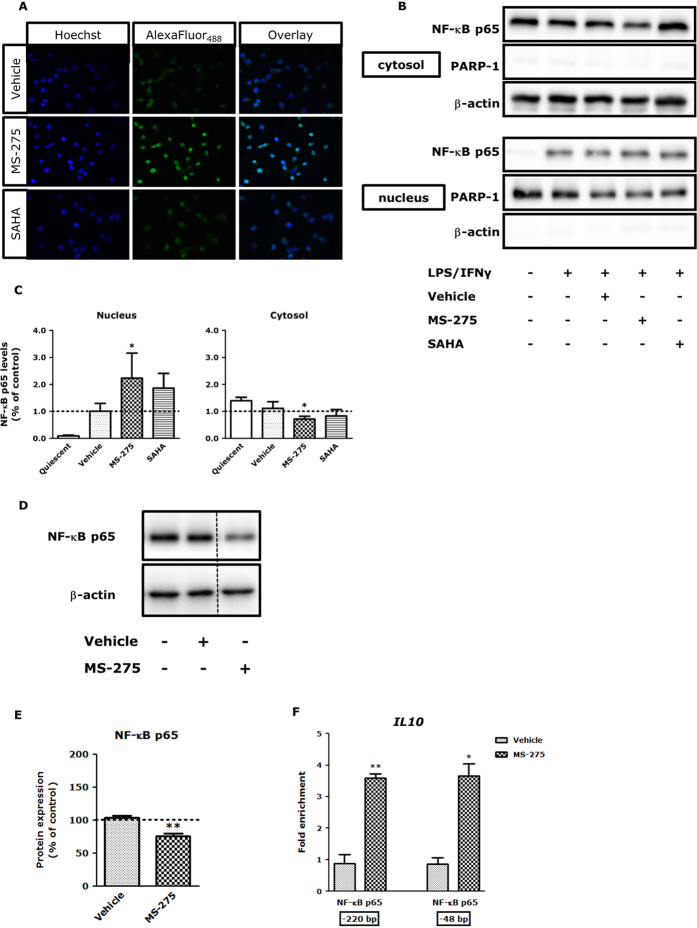
MS-275 enhances the nuclear translocation of NF-κB p65 and enrichment of NF-κB p65 at the transcription start site of *IL10* but reduces total NF-κB p65 protein content in LPS/IFNγ-stimulated RAW264.7 macrophages. After 20 h incubation with MS-275 (1 μM) followed by 1 h LPS/IFNγ stimulation, RAW264.7 macrophages were prepared for immunofluorescence microscopy (**A**). Green signal represents NF-κB p65 protein, while blue signal visualizes the Hoechst-stained nuclei. MS-275 enhanced the translocation of NF-κB p65 compared to vehicle (LPS/IFNγ and inhibitor solvent-treated) cells, while SAHA (0.41 μM) did not affect the nuclear translocation of NF-κB p65. The presented data are representative images of 3 independent experiments, original magnification 400x. All images were taken under identical instrumental conditions. The effect of MS-275 on NF-κB p65 translocation in LPS/IFNγ-stimulated RAW264.7 macrophages was also analyzed by immunoblotting cell fractions (the blots were cropped; full length blots are shown in Figure S6) (**B**) and quantified by densitometry (**C**). As controls for the fractionation PARP-1 (nucleus) and β-actin (cytosol) were used. Data are presented as mean ± SD expressed as fold change compared to control (LPS/IFNγ-treated) cells of 4–5 independent experiments. *p < 0.05 compared to vehicle (LPS/IFNγ and inhibitor solvent-treated) cells. Effects of MS-275 on total NF-κB p65 protein expression in LPS/IFNγ-stimulated RAW264.7 macrophages were analyzed by Western blot (dotted line indicates crop within the same blot; full length blots are shown in Figure S7) (**D**) and quantified by densitometry (**E**). Protein levels were normalized against β-actin and control (LPS/IFNγ-treated) cells were set at 100%. Data are presented as mean ± SD of 4 independent experiments and a representative blot is shown in (**D**). **p < 0.01 compared to vehicle (LPS/IFNγ and inhibitor solvent-treated) cells. qChIP was performed on LPS/IFNγ-stimulated RAW264.7 macrophages treated with MS-275 or vehicle (inhibitor solvent) for 20 h with antibodies against NF-κB p65 (**F**). Enrichment for *IL10* regions −48 bp and −220 bp relative to the transcription start site was analyzed by qPCR. Data are presented as mean ± SEM of 2–3 independent experiments and represent fold enrichment compared to normal rabbit IgG (rIgG). *p < 0.05; **p < 0.01 compared to rIgG.

Effects of MS-275 on NF-κB p65 nuclear translocation were studied using immunofluorescence microscopy. Interestingly, MS-275 increased NF-κB p65 nuclear localization (Fig. 4A), which was confirmed by cellular fractionation experiments in which the nuclear and cytosolic fractions were separated and blotted for NF-κB p65 (Fig. 4B and C). SAHA showed no effect (Fig. 4A–C). In addition, the total NF-κB p65 protein content was studied by Western blot (Fig. 4D) and quantified by densitometry (Fig. 4E). Remarkably, total NF-κB p65 protein content was significantly reduced by 30% in LPS/IFNγ-stimulated RAW264.7 macrophages treated with MS-275 (Fig. 4E). These experiments demonstrate that MS-275 increases the nuclear translocation of NF-κB p65 but decreases total NF-κB p65 content.

To investigate whether NF-κB p65 acts on the *IL10* promoter, chromatin immunoprecipitation (ChIP) was performed. Indeed, a significant increase in enrichment of NF-κB p65 was found around the *IL10* transcription start site (−48 bp and −220 bp upstream) upon MS-275 treatment (Fig. 4F). This indicates that the enhanced activity and nuclear localization of NF-κB p65 in combination with the observed changes in NF-κB and histone acetylation result in increased NF-κB p65 binding to the *IL10* promoter, which is a mechanism that could explain increased *IL10* expression upon MS-275 treatment.

### MS-275 robustly attenuates inflammatory gene expression and inflammatory cell recruitment in cigarette smoke-induced neutrophilic airway inflammation in C57Bl/6 mice

**Figure 5:**
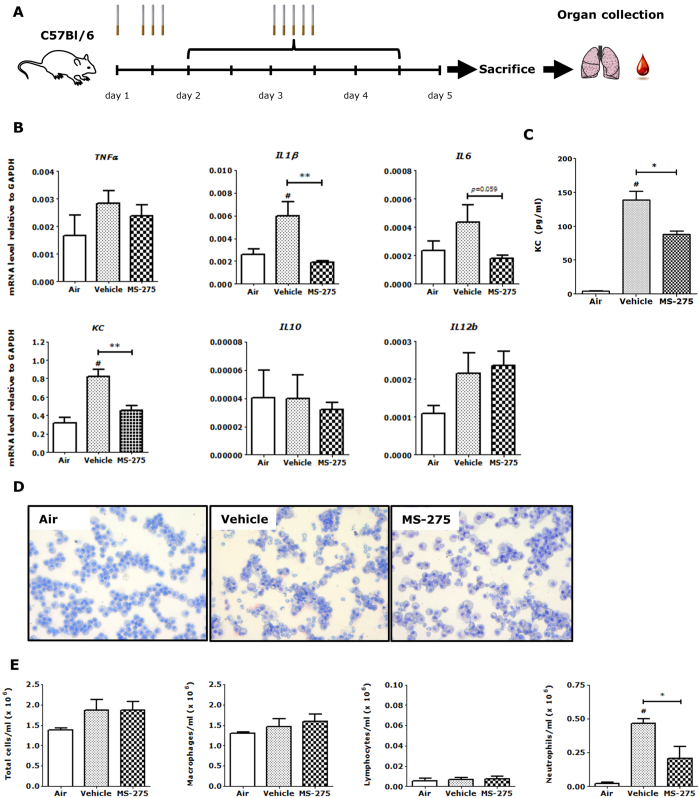
Attenuation of cigarette smoke-induced neutrophilic airway inflammation by MS-275. Experimental procedure (**A**). Male C57Bl/6 mice were exposed to cigarette smoke twice daily on four consecutive days by whole body exposure. Mice were subjected to MS-275 (10 μM by nebulization) or vehicle (inhibitor solvent) 30 min prior to each cigarette smoke exposure. Bronchoalveolar lavage (BAL) was performed and lungs were collected 16 h after the last smoke exposure. Gene expression in lung homogenates was studied by RT-qPCR and expressed relative to GAPDH (**B**). MS-275 robustly reduced *IL1β* and *KC* mRNA levels in cigarette smoke exposed mice. Data are presented as mean ± SD; n = 4–5 animals per group. **p < 0.01 compared to vehicle (smoke and inhibitor solvent-treated) group ^#^*p* < 0.05 compared to air-exposed group. Release of KC in BAL fluid was determined by ELISA (**C**). MS-275 significantly reduced KC release in BAL fluid of cigarette-smoke exposed mice. Data are presented as mean ± SD; n = 4–5 animals per group. *p < 0.05 compared to vehicle (smoke and inhibitor solvent-treated) mice. Inflammatory cell count was performed in BAL fluid **(D**) and total cells, macrophages, lymphocytes and neutrophils were plotted as number of cells/ml (**E**). Total cell number in cigarette smoke exposed mice was not affected by MS-275. Neutrophilic infiltration was reduced by 60% in MS-275-treated mice compare to vehicle (smoke and inhibitor solvent-treated) mice. Data are presented as mean ± SD; n = 4–5 animals per group. *p < 0.05 compared to vehicle (smoke and inhibitor solvent-treated) mice.

To shed more light on the mixed effects observed on pro- and anti-inflammatory gene expression in RAW264.7 macrophages and PCLS upon MS-275 treatment, studies were continued to elucidate whether *in vivo* lung inflammation could be dampened by MS-275 in an acute cigarette smoke-induced neutrophilic airway inflammation model in C57Bl/6 mice. For a validation of the transition from the LPS/IFNγ to cigarette smoke exposure, RAW264.7 macrophages were exposed to cigarette smoke extract (CSE). Increased *IL10* expression by MS-275 was also found under these conditions (Figure S8). Mice were pre-treated with MS-275 prior to each cigarette smoke exposure using an experimental setup shown in Fig. 5A.

To study if MS-275 affects liver or kidney function in the mice, liver and kidney function parameters were analyzed (Table S2). No changes were observed. Also, no changes in the weight of the animals were observed (Figure S9). These data show that 10 μM MS-275 does not affect liver and kidney function, or animal weight.

Expression levels of the inflammatory genes *TNFα, IL1β, IL6, KC (murine IL8), IL12b* and the anti-inflammatory gene *IL10* were examined in lung tissue homogenates. Cigarette smoke exposure significantly increased expression of *IL1β* and *KC* compared to air-exposed mice. MS-275 treatment resulted in a robust downregulation of *IL1β* and *KC* (Fig. 5B). An almost significant decrease was observed for *IL6* expression (*p = *0.059). While not significant, *IL6* expression was reduced to levels observed in air-exposed mice, similar to *IL1β* and *KC*. MS-275 treatment did not affect expression of *TNFα, IL12b* or *IL10* in whole lung tissue homogenates of cigarette smoke-exposed mice. The level of *p65* was also measured but remained unchanged in the lung tissue homogenates (Figure S10). The lack of effect on IL10 and p65 expression could indicate that effects of MS-275 on their expression are difficult to measure due to the presence of many different cell types in lung tissue. This stresses the need for studying specific cell types such as macrophages. Taken together this shows that MS-275 attenuates inflammatory gene expression in a cigarette smoke-induced neutrophilic airway inflammation model in C57Bl/6 mice.

The acute cigarette smoke exposure model in C57Bl/6 mice is characterized by neutrophil infiltration. Neutrophil infiltration is also increased in patients with COPD and correlates with disease severity^33^. The increase in neutrophils is related to increased production of CXC-chemokines such as CXCL8 (also known as IL-8). IL-8 attracts neutrophils, since it is a ligand for the CXCR2 receptor which is expressed predominantly by neutrophils^33^. The mouse orthologue of IL-8 is keratinocyte-derived chemokine (KC). Therefore, KC release in bronchoalveolar lavage (BAL) fluid was determined. Levels of KC in BAL fluid of cigarette smoke-exposed mice were 30-fold higher compared to air-exposed mice (Fig. 5C). Interestingly, in line with the observed reduction of *KC* mRNA in whole lung tissue homogenates, KC release in cigarette smoke-exposed mice pre-treated with MS-275 was significantly reduced by 40% (Fig. 5C). To study the effect of MS-275 on cigarette smoke-induced inflammatory cell recruitment, cell counts were determined in BAL fluid. Mice exposed to cigarette smoke had an increased number of inflammatory cells compared to air-exposed animals (Fig. 5D and E). The predominant cell type in the BAL fluid was the macrophage, which was comparable between air-exposed and cigarette smoke-exposed mice. Neutrophilic infiltration was increased by 20-fold in cigarette smoke-exposed compared to air-exposed mice. In line with the observed reduction of *KC* mRNA in lung tissue homogenates and KC cytokine in BAL, a 55% decrease in neutrophil number was observed in cigarette smoke-exposed mice pre-treated with 10 μM MS-275 compared to cigarette smoke-exposed mice subjected to vehicle (Fig. 5D and E). Taken together, MS-275 reduces the KC release in BAL fluid of cigarette smoke-exposed mice, which correlates with inhibition of neutrophilic lung infiltration. These data demonstrate for the first time that MS-275 attenuates inflammatory responses in cigarette smoke-induced neutrophilic airway inflammation in mice *via* a mechanism in which the KC cytokine release is attenuated, which correlates with robust attenuation of neutrophilic influx.

### MS-275 restores mRNA levels of IL-10 *in vivo* but does not affect the CD68^+^ or CD68^+^/IL-10^+^ cell number in cigarette smoke-induced C57Bl/6 mice

**Figure 6:**
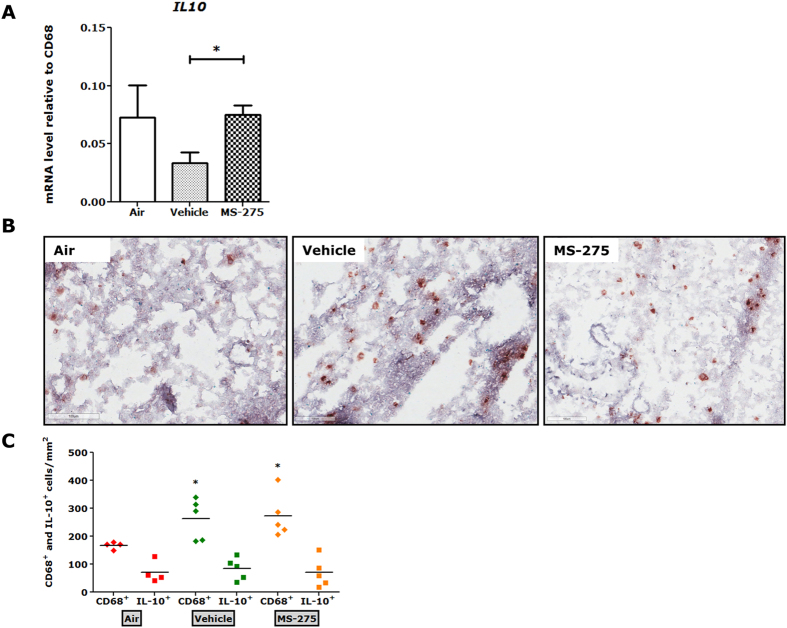
MS-275 restored the *in vivo* mRNA levels of *IL10* in macrophages but did not affect the CD68^+^ or CD68^+^/IL-10^+^ cell number in lungs of cigarette smoke-exposed mice. MS-275 restored *IL10* mRNA levels of cigarette smoke-exposed mice to mRNA levels of air-exposed mice (**A**). Data are presented as means ± SD; n = 4–5 animals per group. *p < 0.05 compared to vehicle (smoke and inhibitor solvent-treated) mice. Numbers of CD68^+^ and CD68^+^/IL-10^+^ macrophages in the lungs were determined by immunohistochemistry (**B**). Images were taken with identical instrumental conditions and representative images of 4–5 independent mice per group are shown (original magnification 200x). Immunohistochemical stainings were quantified manually and presented as number/mm^2^ lung tissue (**C**). Scatter plots showing an increase in CD68^+^ macrophages upon cigarette smoke exposure. CD68^+^/IL-10^+^ macrophages remained unaffected. MS-275 did not affect the influx of CD68^+^ or CD68^+^/IL-10^+^ macrophages. Data are presented as mean ± SD; n = 4–5 animals per group. *p < 0.05 compared to air-exposed animals.

MS-275 increased *IL10* levels in RAW264.7 macrophages (Fig. 2). Therefore, we hypothesized that the anti-inflammatory effects of MS-275 in cigarette smoke-induced C57Bl/6 mice could be mediated by increased numbers of IL-10^+^ macrophages in the lungs. We studied the mRNA expression of *IL10* in lung tissue of mice exposed to cigarette smoke and pre-treated with MS-275, relative to *CD68* (macrophage marker). Interestingly, in cigarette smoke-exposed mice pre-treated with MS-275, mRNA expression of *IL10* relative to *CD68* was re-established to levels observed in air-exposed mice (Fig. 6A). This suggests that MS-275 restores the ability of cigarette smoke-exposed macrophages to produce IL-10. Next, we studied if the re-established *IL10* mRNA level affects the number of IL-10^+^ macrophages. Lung cryosections were immunohistochemically stained for CD68^+^ and IL-10 (Fig. 6B). Cigarette smoke exposure increased the CD68^+^ cell number compared to lung tissue of air-exposed mice (Fig. 6C). However, MS-275 did not affect the CD68^+^ or the CD68^+^/IL-10^+^ cell number in cigarette smoke-exposed mice (Fig. 6C). These data do not support the hypothesis that the observed anti-inflammatory effects of MS-275 in cigarette smoke-induced C57Bl/6 mice are mediated by increased numbers of CD68^+^/IL-10^+^ macrophages.

### MS-275 restores overall histone acetylation in lung tissue of cigarette smoke-exposed mice

**Figure 7:**
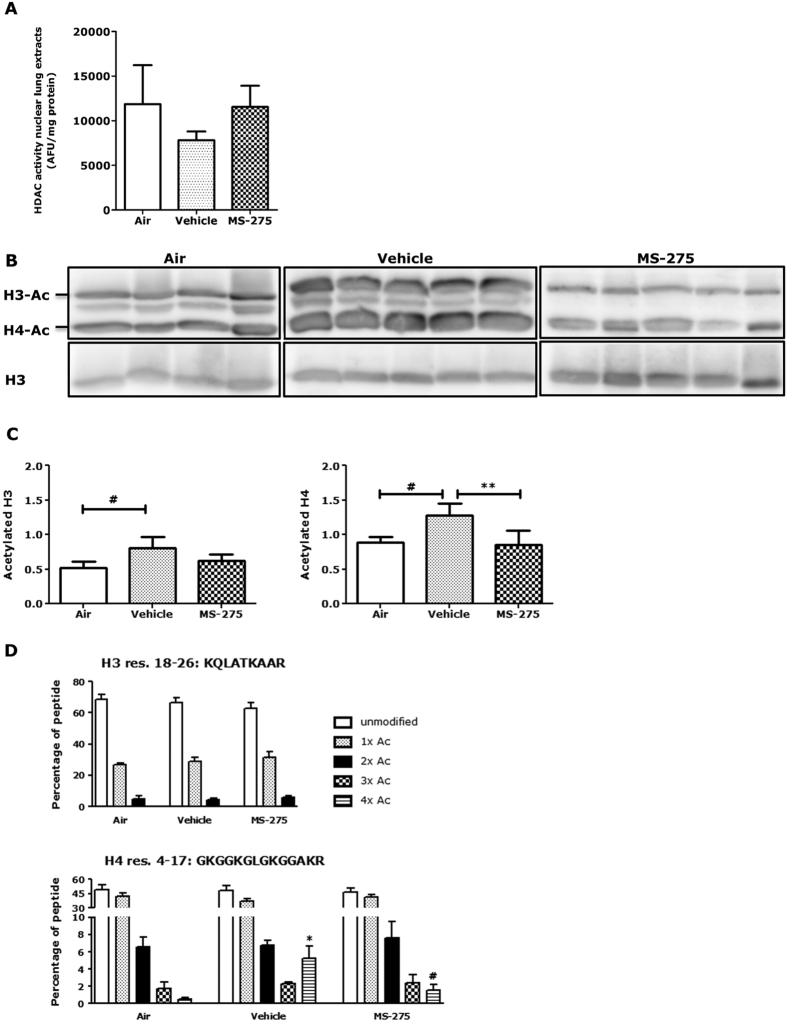
MS-275 reduced acetylation of histone H3 and H4 in cigarette smoke-exposed mouse lungs. To study the effect of MS-275 on total HDAC activity in nuclear lung extracts of cigarette smoke-exposed mice, HDAC activity was determined based on the conversion of a pro-fluorogenic substrate **(A**). MS-275 restored HDAC activity compared to air-exposed mice. Data are presented as mean ± SD of 4–5 animals per group. Effects of MS-275 on histone H3 and H4 acetylation were studied by Western blot (blots were cropped; full length blots are shown in Figure S11 **(B)** and quantified by densitometry **(C)**. Total histone H3 and H4 acetylation was increased in cigarette smoke-exposed mice lungs, and for histone H4 this was reduced by MS-275. ^#^*p* < 0.05 compared air-exposed animals **p < 0.01 compared to vehicle (smoke and inhibitor-solvent treated) animals. Histone acetylation of histone H3 and H4 was also analyzed by mass spectrometry **(D)**. Histones were resolved by SDS-PAGE, histones H3 and H4 were excised from the gel and subjected to LC-MS/MS analysis. Smoke exposure increased, and MS-275 reduced, acetylation on one peptide from histone H4 (res. 4–17: GKGGKGLGKGGAKR). Other peptides were not affected. Data are presented as mean ± SD of 4–5 animals per group. *p < 0.05 compared to air-exposed animals. ^#^*p* < 0.05 compared to vehicle (smoke and inhibitor-solvent treated) animals.

We anticipated that in mice treatment with MS-275 would result in decreased HDAC activity and increased histone acetylation. Thus, HDAC activity was determined in lung tissue homogenates using an HDAC activity assay employing the conversion of a pro-fluorogenic substrate as described previously^34^. Total HDAC activity was reduced in cigarette smoke-exposed mice compared to air-exposed mice (Fig. 7A). Although not significant due to relatively large standard deviations, remarkably, total HDAC activity appeared to be re-established in cigarette smoke-exposed mice pre-treated with MS-275 compared to air-exposed mice. Subsequently, histone acetylation was studied by Western blot. In line with previous studies in cells^35^, acetylation of both histone H3 and H4 was significantly increased in lung tissue of cigarette smoke-exposed mice compared to air-exposed mice. Excitingly, a decrease in H4 acetylation was observed upon MS-275 treatment (Fig. 7B), which was confirmed by densitometry (Fig. 7C). Acetylation of histone H3 and H4 was then analyzed using mass spectrometry. In line with the Western blots, decreased acetylation upon MS-275 treatment was observed on res. 4–17 from histone H4 (Fig. 7D). Other histone peptides were not affected. Altogether, this shows for the first time that histone H4 acetylation is increased in cigarette smoke-induced C57Bl/6 mice, which is attenuated by MS-275.

## Discussion

We studied the HDAC1-3-selective inhibitor MS-275 in *ex vivo, in vitro* and *in vivo* COPD models, to explore potential therapeutic applications. While mixed effects were observed in PCLS and RAW264.7 macrophages, in cigarette smoke-exposed C57Bl/6 mice there were robust anti-inflammatory effects.

Although MS-275 increased the expression of pro-inflammatory genes in PCLS (except *TNFα*) and RAW264.7 macrophages, importantly, the anti-inflammatory expression of *IL10* was upregulated. This may be particularly relevant in COPD, based on numerous examples. IL-10 can inhibit LPS-induced *TNFα* and *IL8* expression^36^. Interstitial macrophages producing IL-10 negatively regulate T helper 2 and 17 cell-mediated inflammatory responses, which prevents neutrophilic asthma^37^. In COPD patients, the IL-10 concentration in serum and sputum was decreased compared to non-smokers^38^. In lung tissue of COPD patients less IL-10 was found upon LPS stimulation than in lung tissue of patients with normal lung function^39^. Macrophages in COPD have lost the ability to produce anti-inflammatory cytokines like IL-10, and therefore cannot effectively dampen expression^39^,^40^. In summary, macrophages play an important role in COPD, and IL-10 can determine the functional role of macrophages. Restoring *IL10* expression could be relevant in resolving inflammation in COPD.

Effects of MS-275 on inflammatory gene expression in macrophages have been described before. MS-275 was previously shown to upregulate IL-10 protein level in RAW264.7 macrophages^25^, but opposite to our observations, IL-1β was downregulated. MS-275 also reduced nitric oxide (NO) concentration in the media and decreased mRNA levels of *iNOS* and *TNFα*, showing anti-inflammatory effects^25^,^41^. MS-275 reduced LPS-induced IL-1β, IL-6, IL-18 and TNF-α secretion in THP-1 human monocytic cells; and NO secretion in RAW264.7 cells^23^. In bone marrow derived macrophages MS-275 showed mainly pro-inflammatory effects on gene expression^42^. MS-275 reduced poly(I-C)- induced release of TNF-α, IL-6, and IL-12 in dendritic cells. However, IL-10 was also reduced, indicating a mixed pro- and anti-inflammatory effect opposite to our findings (i.e. downregulation instead of upregulation)^43^. Thus, MS-275 gives rise to either pro- or anti-inflammatory effects in these cells. The exact experimental conditions are likely important to the outcome. Furthermore, future studies could include an assessment of the metabolism of macrophages to distinguish between different macrophage phenotypes, as a mixed population of macrophages could also explain these effects.

The molecular mechanism underlying the increased *IL10* expression under the conditions applied in this study was studied in macrophages, focusing on NF-κB as a potential regulator. MS-275 increased NF-κB promoter activity, acetylation, and nuclear translocation in RAW264.7 macrophages. The total NF-κB p65 protein level was reduced. Importantly, MS-275 increased NF-κB p65 binding to the *IL10* promoter. This mechanism is in line with literature^32^. MS-275 was also previously shown to increase NF-κB p65 nuclear translocation, DNA binding, acetylation, and activity in U937 human monocytes. The total p65 level was unchanged^44^. In mouse primary cortical neurons MS-275 increased global NF-κB acetylation^45^. In contrast, MS-275 was previously shown to inhibit LPS-induced NF-κB p65 nuclear accumulation and acetylation^23^. Taken together, this shows that the effects of MS-275 on NF-κB p65 are complex. The different observations for effects of MS-275 on NF-κB p65 could be explained by the different concentrations of MS-275 that were used, different time points used, and differences in the employed inflammatory stimulus. Also, effects may vary between cell types.

SAHA did not affect gene expression, NF-κB p65 acetylation or translocation in macrophages, while a robust increase in histone acetylation was observed. These effects are distinct from those of MS-275. Thus, under the applied conditions, iso-enzyme selectivity of HDACi has important consequences for their effects. The iso-enzyme selectivity of MS-275, which inhibits HDAC1 and 2, and 3, is also important considering the role of HDAC2 in COPD. HDAC2 expression and activity are decreased in COPD, which is linked to steroid resistance^46^. For HDAC1 and 3, roles in COPD have not clearly been described. Altogether this shows that development of highly potent molecules selectively targeting specific HDACs is of interest. This could aid in obtaining selective anti-inflammatory effects *via* regulation of NF-κB acetylation on specific lysines. Based on the important role of HDAC3 in deacetylation of specific NF-κB p65 lysines^18^, HDAC3-selective inhibitors may be interesting candidates for studies in mice. In support of this, the HDAC3-selective inhibitor RGFP966 was previously shown by us to increase *IL10* expression and reduce pro-inflammatory gene expression in PCLS. In RAW264.7 macrophages, pro-inflammatory gene expression was decreased, and correlated with reduced NF-κB activity^47^. This is particularly interesting considering the increased NF-κB activity reported in COPD^8^.

Nevertheless, importantly, in cigarette smoke-exposed C57Bl/6 mice, MS-275 attenuated inflammatory responses by robustly decreasing KC cytokine release and neutrophil influx. In line with this, reduced inflammatory responses have been reported in various animal disease models characterized by inflammation^24^,^25^,^48^,^49^. This shows that in cigarette-smoke-exposed C57Bl/6 mice, but also in other disease models, MS-275 can suppress inflammatory responses. This indicates the potential for development of selective HDACi for treatment of inflammatory (lung) diseases.

Considering the increased *IL10* expression in PCLS and RAW264.7 macrophages, we expected that MS-275 also increases *IL10* expression in macrophages *in vivo*, playing a role in the anti-inflammatory effects. Indeed, MS-275 restored mRNA levels of *IL10* in macrophages in lungs from cigarette smoke-exposed mice to levels of macrophages in air-exposed mice. Despite this restoration, the CD68^+^/IL10^+^ cell number was unchanged. However, even though cell numbers were not increased, IL-10 levels could still have been increased. Previously, MS-275 was shown to increase *IL10* expression *in vivo* in lymph nodes of experimental autoimmune neuritis rats, and increase the proportion of macrophages expressing anti-inflammatory cytokines (compared to pro-inflammatory cytokine expressing macrophages)^24^. MS-275 also increased the proportion of macrophages expressing anti-inflammatory cytokines in prostates of experimental autoimmune prostatitis rats^25^. Our results and these studies combined suggest that MS-275 can increase IL-10 levels in macrophages *in vivo*, and lead to a (relative) increase in macrophages with an anti-inflammatory profile, in several rodent disease models characterized by inflammation.

The anti-inflammatory effects *in vivo* were not directly reflected in PCLS and RAW264.7 macrophages, where MS-275 increased expression of both pro- and anti-inflammatory genes. It is unclear why this is the case. Although our results in macrophages *in vitro* clearly indicate a mechanism in which MS-275 increases *IL10* expression by increasing NF-κB p65 activity and *IL10* promoter binding, it is possible that MS-275 shows anti-inflammatory effects *in vivo* through other mechanisms. Nevertheless, we anticipate that the identified molecular mechanism could be one of the contributors. A possible future study to confirm this would be to assess isolated macrophages from smoke exposed mice or clinical COPD samples.

As expected, MS-275 increased histone acetylation in PCLS and RAW264.7 macrophages. In line with previous studies in cells^35^, cigarette smoke increased histone H4 acetylation in mouse lungs. Strikingly, and counter-intuitively, MS-275 reduced these acetylation levels. This suggests that the overall changes in histone acetylation are a consequence of processes brought about by cigarette smoke exposure, which are alleviated by MS-275; rather than a direct consequence of MS-275 treatment. The observations of less KC (murine IL-8) secretion and neutrophil influx upon MS-275 treatment could be accompanied by reduced histone acetylation. This could be explained by increased IL-10 secretion by alveolar macrophages. In monocytes IL-10 has been described to locally decrease histone H4 acetylation at the *IL8* promoter^36^. Compensation mechanisms could also take place upon MS-275 treatment to restore overall HDAC activity resulting in reduced acetylation levels. Previously, MS-275 increased histone acetylation in various tissue types *in vivo* in other disease models^50^,^51^,^52^. It is unclear why our results are not in line with this, and this requires further study.

In summary, MS-275 showed mixed effects on pro- and anti-inflammatory gene expression in PCLS and RAW264.7 macrophages. Importantly, anti-inflammatory *IL10* expression was upregulated in these models. In macrophages this was connected to increased NF-κB p65 transcriptional activity, acetylation, nuclear localization, and binding to the *IL10* promoter. This molecular mechanism could be relevant to COPD due to the crucial role of macrophages and IL-10 in COPD. Importantly, in cigarette smoke-induced C57Bl/6 mice, MS-275 reduced inflammatory gene expression of *KC* mRNA, KC cytokine level in BAL, and lung neutrophil influx. *IL10* expression was upregulated *in vivo* in lung macrophages on the mRNA but not the protein level. Intriguingly, MS-275 decreased histone acetylation in the lungs of cigarette smoke-exposed mice. To our knowledge, this study is the first to demonstrate that the HDAC1-3-selective inhibitor MS-275 robustly attenuates cigarette smoke-induced neutrophilic airway inflammation in C57Bl/6 mice. This highlights a potential alternative application area for isoform selective HDACi in the treatment of inflammatory lung diseases, which is a novel concept.

## Materials and Methods

### HDAC inhibition assay

The HDAC inhibition assay was performed in triplicate as described before^34^. Briefly, the respective human recombinant HDAC enzymes (BPS Bioscience, San Diego, CA, USA) were incubated in absence and/or in presence of various concentrations MS-275, SAHA and the pro-fluorogenic substrate at room temperature for 60 min. Next, the deacetylation reaction was stopped with the addition of the HDAC Stop Solution (6 mg/mL trypsin, 0.3 mM SAHA) in all wells and the plate was incubated at 37 °C for 20 min. The release of the fluorescent 7-amino-4-methylcoumarin was monitored by measuring the fluorescence at λ_em_ = 460 nm and λ_ex_ = 390 nm using a Synergy H1 plate reader (BioTek Instruments, Winooski, VT, USA). The fluorescence value of the background wells was subtracted from the fluorescence of the positive control, blank and inhibitor wells. Nonlinear regression was used to fit the data to the log(inhibitor) versus response curve using GraphPad Prism (GraphPad software 5.00, San Diego CA, USA).

### Precision-cut lung slices and treatment

C57bl/6 male mice (weight 24–28 g; age 8–10 weeks) were purchased from Harlan (Zeist, The Netherlands) and maintained on mouse chow and tap water *ad libitum* in a humidity- and temperature-controlled room at 24 °C with a 12 h light/dark cycle. All experiments were performed according to national guidelines and upon approval of the experimental procedures by the local Animal Care and Use committee of Groningen University, DEC number 6962 A. Mice were randomly assigned to the experiments.

Mouse precision-cut lung slices (PCLS) were prepared as previously described^53^. Briefly, male mice were anesthetized by subcutaneous injection of ketamine (40 mg/kg, Alfasan, Woerden, The Netherlands) and dexdomitor (0.5 mg/kg, Orion Pharma, Mechelen, Belgium). Subsequently, the trachea was cannulated and the animal was exsanguinated by cutting the jugular vein, after which the lungs were filled trough the cannula with 1.5 mL low melting-point agarose solution (1, 5% final concentration) The lungs were placed on ice for 15 min to solidify the agarose for slicing. The lobes were separated and tissue cores were prepared of the individual lobes, after which the lobes were sliced at a thickness of 250 μm. Tissue slices were incubated at 37 °C in a humid atmosphere under 5% CO_2_/95% air. In order to remove the agarose and cell debris from the tissue, slices were washed every 30 min (four times in total).

PCLS were incubated in DMEM supplemented with sodium pyruvate (1 mM), MEM non-essential amino acid mixture (1:100; Gibco^®^ by Life Technologies), gentamycin (45 μg/mL; Gibco^®^ by Life Technologies), penicillin (100 U/mL), streptomycin (100 μg/mL) and amphotericin B (1.5 μg/mL; Gibco^®^ by Life Technologies). Slices were cultured at 37 °C in a humidified atmosphere under 5% CO_2_/95% air in 12-well tissue culture plates, using 3 slices per well. Slices were treated with MS-275 (1 and 10 μM) and SAHA (0.41 μM) for 20 h and where indicated, stimulated with 10 ng/mL LPS/IFNγ.

### Cell culture

RAW264.7 macrophages (mouse) were obtained from the American Type Culture Collection (ATCC, Wesel, Germany) and cultured in plastic tissue culture plates or flasks (Costar Europe, Badhoevedorp, The Netherlands) at 37 °C under 5% CO_2_/95% air in Dulbecco’s Modification of Eagle’s Medium (DMEM) containing GlutaMAX^TM^ (Gibco^®^ by Life Technologies, Bleiswijk, The Netherlands) supplemented with 10% (v/v) heat inactivated fetal bovine serum (FBS; Invitrogen, Breda, The Netherlands), 2 mM additional GlutaMAX^TM^ (Gibco^®^ by Life Technologies), 100 U/mL penicillin (Gibco^®^ by Life Technologies) and 100 μg/mL streptomycin (Gibco^®^ by Life Technologies). RAW264.7 macrophages were used between passage 5 and 16.

Where indicated, cells were stimulated with 10 ng/mL lipopolysaccharide (LPS, *Escherichia coli*, serotype 0111:B4; Sigma-Aldrich, Zwijndrecht, The Netherlands) and 10 ng/mL interferon gamma (IFNγ, #315-05; PeproTech, Hamburg, Germany) for the last 4 h of the experiment. For exposure to cigarette smoke extract (CSE), RAW264.7, 5% CSE was used. CSE was prepared by a standardized method by combusting two 3R4F research cigarettes (University of Kentucky, Lexington, KY) (filter removed) using a peristaltic pump (Watson Marlow, Falmouth, Cornwall, England) and passing the smoke through 25 ml of the culture medium, at a rate of one cigarette per 5 min. The obtained solution was designated 100% CSE and diluted to working concentration in the culture medium. For all experiments, CSE was freshly prepared and used within 30 min after preparation.

### Investigation of cellular toxicity by MTS assay

To investigate the influence of the HDACi on cell viability, RAW264.7 macrophages were seeded in 96-well plates. To obtain identical cell density at the start of the experiments, RAW264.7 macrophages were seeded at 25,000 cells/cm^2^, prior incubation with MS-275 and SAHA. Shortly before incubation with HDACi, medium was replaced by 100 μL fresh culture medium. After 20 h of incubation with HDACi (and if appropriate the last 4 h supplemented with 10 ng/mL LPS/IFNγ), 20 μL of CellTiter 96 AQ_ueous_ One Solution reagent (Promega) was added to each well and incubated at 37 °C for 1 h in the dark. The absorbance at 490 nm was measured using a Synergy H1 plate reader. LPS/IFNγ-stimulated cells without addition of HDACi were considered 100%.

### Gene expression analysis by RT-qPCR

RAW264.7 macrophages were washed twice with ice-cold Dulbecco’s Phosphate-buffered Saline (DPBS, Gibco^®^ by Life Technologies) and total RNA was isolated using the SV Total RNA Isolation kit (Promega, Leiden, The Netherlands). RNA from mouse precision-cut lung slices (PCLS) and from mouse lung tissue (whole lung) was isolated using the Maxwell^®^ 16 LEV simplyRNA Tissue Kit (Promega). Both RNA isolation procedures were performed according to the manufacturer’s protocol. RNA integrity was determined by 28 S/18 S ratio detection on an 1% agarose gel, which was consistently found intact. RNA concentration (OD260) and purity (OD260/OD280) were measured by NanoDrop ND-1000 UV-Vis spectrophotometer (NanoDrop Technologies, Wilmington, DE, USA). For gene expression analysis, RNA was reverse transcribed using the Reverse Transcription kit (#A3500, Promega). Subsequently, 10 ng of cDNA was applied for each RT-qPCR, which was performed on an ABI Prism 7900HT Sequence Detection System (Applied Biosystems, Nieuwerkerk a/d IJssel, The Netherlands). The primers for TNFα (Mm00443258_m1, IL-1β (Mm00434228_m1), IL-6 (Mm00446190_m1), KC (Mm04208136_m1), IL-10 (Mm00439614_m1), IL-12b (Mm00434174_m1), CD68 (Mm03047340_m1), p65 (Mm00501346) and GAPDH (Mm99999915_g1) were purchased as Assay-on-Demand (Applied Biosystems). For each sample, the real-time PCR reactions were performed in duplicate and the averages of the obtained C_t_ values were used for further calculations. Data analysis was performed with the relative quantification manager software (SDS 2.4; Applied Biosystems). Gene expression levels were normalized to the expression of the reference gene glyceraldehyde-3-phosphate dehydrogenase (GAPDH), which was not influenced by the experimental conditions resulting in the ΔC_t_ value. Gene expression levels were calculated by the comparative C_t_ method (2^−ΔΔCt^)^54^.

### Assessment of tissue viability using lactate dehydrogenase

To assess the viability of the PCLS subjected to MS-275 and SAHA, the amount of lactate dehydrogenase (LDH) released from the tissue slices into the incubation medium was analyzed. Maximal LDH release was determined by lysing 3 slices with 1% Triton X-100 for 30 min at 37 °C at the start of the experiments. Supernatants were stored at −80 °C. LDH release was determined using an assay form Roche Diagnostics (Mannheim, Germany), and was measured using a Hitachi automatic analyzer (Modular Analytics, Roche Diagnostics). LDH release from the PCLS into the incubation medium was plotted relative to maximal LDH release.

### Protein expression analysis by Western blot

RAW264.7 macrophages were washed twice with ice-cold DPBS and subsequently lysed in ice-cold cell lysis buffer (25 mM Hepes, 5 mM MgCl_2_, 5 mM EDTA, 0.5% Triton X-100; supplemented with 1 mM DTT, 1 mM sodium butyrate, and protease inhibitors (#88266; Thermo Scientific, Rockford, IL, USA)). Next, lysates were freeze-thawed (4x) and centrifuged (10 min, 13,000 g) to remove cell debris. Protein concentrations were determined using the RC DC Protein Assay (Bio-Rad) according to the manufacturer’s protocol. Equal amounts of protein were loaded on a 10% polyacrylamide gel, separated by SDS-PAGE with a Mini**-**Protean II apparatus (Bio-Rad Laboratories, Veenendaal, The Netherlands), and transferred with a Trans-Blot Electrophoretic Transfer system (Bio-Rad Laboratories) onto a polyvinylidene difluoride membrane (PVDF; Bio-Rad Laboratories). The membrane was blocked at room temperature for 1 h in DPBS/0.1% Tween 20 (Sigma-Aldrich; solution referred to as PBST) containing 5% skimmed milk (Campina, Friesland, The Netherlands) and subsequently incubated overnight at 4 °C with the appropriate primary antibody in 5% BSA (Sigma-Aldrich) or 5% skimmed milk in PBST. The following primary antibodies and dilutions were used: NF-κB p65 (#8242, 1:2,500), PARP-1 (#9532, 1:1,000), and Histone H3 (#4499, 1:2,000); all from Cell Signaling, Leiden, The Netherlands and anti-acetyl lysine (AB3879, 1:500; Millipore, Billerica, MA, USA). For loading control the lower part of the blot was incubated for 1 h at room temperature with monoclonal rabbit anti-β-actin (#4970, 1:10,000; Cell Signaling). Membranes were washed in PBST and incubated at room temperature for 1 h with peroxidase-conjugated secondary antibodies. The following secondary antibodies were used: goat anti-rabbit IgG/HRP (#P0448), rabbit anti-goat IgG/HRP (#P0449) and rabbit anti-mouse IgG/HRP (#P0260, all 1:2,000; DakoCytomation, Glostrup, Denmark). The bands were visualized using the VisiGlo^TM^ Prime HRP Chemiluminescenct Substrate Kit (AMRESCO, Solon, OH, USA), quantified by imaging (GeneSnap from SynGene, Frederick, MD, USA), and processed using ImageJ software (1.48d; National Institute of Health, USA). For the Western blot of K310 acetylated p65, RAW264.7 macrophages (from one 175 cm^2^ culture flask) were subjected to MS-275 (1 μM) for 20 h, stimulated with 10 ng/mL LPS/IFNγ for 4 h and subsequently lysed in lysis buffer (20 mM Tris-HCl pH 7.5, 120 mM NaCl, 1% IGEPAL CA-630, 2 mM EDTA; all from Sigma-Aldrich) supplemented with Pierce^TM^ Protease Inhibitors (P.I.; #88266, Thermo Scientific). The lysates were loaded on a 10% polyacrylamide gel, and the same blotting and blocking procedures as described above were followed. The primary antibody that was used was #ab19870 from Abcam in 1:500 dilution. The same secondary antibody and visualization procedure as described above was used. The K310 acetylation signal was quantified using the p65 signal as a loading control.

### Effect of HDACi on NF-κB transcriptional activity by QUANTI-Blue assay

RAW-Blue^TM^ cells (InvivoGen, San Diego, CA, USA) were originally derived from mouse RAW264.7 macrophages which stably express a secreted embryonic alkaline phosphatase (SEAP) gene inducible by NF-κB p65 and AP-1 transcription factors. RAW-Blue cells were treated with MS-275 or SAHA for a total of 20 h from which the last 4 h supplemented with 10 ng/mL LPS/IFNγ. The secretion of SEAP into the medium is therefore indicative of NF-κB activity, and was determined using the QUANTI-Blue^TM^ detection medium (InvivoGen) according to the manufacturer’s protocol. Briefly, 50 μL of supernatant was added to 150 μL of QUANTI-Blue^TM^ detection medium and incubated at 37 °C for 1–2 h in the dark. Subsequently, SEAP activity was assessed by measuring the absorbance at 630 nm using an Synergy H1 plate reader. Results were plotted as % of control.

### Histone extraction and Micro BCA™ Protein Assay

Histone extractions were performed as previously described in the literature with minor modifications^55^. After histone extractions the samples were diluted with Phosphate-Buffered Saline (PBS, PAA Laboratories GmbH, Austria) to determine the total protein concentration using the micro BCA protein assay according to the manufacturer’s instructions (Pierce, Rockford, USA, # 23235). Absorbance was measured with a Synergy H1 plate reader at 562 nm. A bovine serum albumin standard (2 mg/mL, Pierce, Rockford, USA, # 23209) was used to calibrate the assay.

### Acetylation of histones with acetic anhydride-d6

For the in-gel reaction of histones with acetic anhydride-d6, 7 μg of histones were loaded on a 15% polyacrylamide gel and resolved by SDS-PAGE electrophoresis. After Coomassie staining, bands for histones H3 and H4 were excised from the gel. Then 50 μL of acetonitrile and 50 μL of ammonium bicarbonate buffer (100 mM) were added to destain the gel bands. Gel bands were then dried in acetonitrile. Subsequently, 5 μL of acetic anhydride-d6 was added, after which 100 μL of ammonium bicarbonate (1 M) was added immediately. Subsequently, samples were incubated at 37 °C for 15 min. Gel bands were then washed 3 times with H_2_O. The acetic anhydride-d6 reaction was then repeated. After the second reaction, gel bands were dried using acetonitrile and trypsin (Promega, Madison, Wisconsin, USA, # V511A) was added at a 1:20 ratio in ammonium bicarbonate (50 mM). Histones were digested at 37 °C for 16 h. Supernatants containing histone peptides were subjected to LC MS/MS analysis as described below.

### NanoChip LC-MS/MS QTOF

A quadrupole time-of-flight mass spectrometer (QTOF, Agilent 6510) with a liquid chromatography-chip cube (# G4240) electrospray ionization interface was coupled to a nanoLC system (Agilent 1200) composed of a nanopump (# G2226A), a capillary loading pump (#G1376A) and a solvent degasser (# G1379B). Injections were performed with an autosampler (# G1389A) equipped with an injection loop of 40 μL and a thermostated cooler maintaining the samples in the autosampler at 4 °C during the analysis (#G1377A Micro WPS). The instrument was operated under the MassHunter Data Acquisition software (Agilent Technologies, Santa Clara, USA, version B.04.00, B4033.3). A chip (ProtID-Chip-150 II 300 A, #G4240-62006) with a 40 nl trap column and a 75 μm × 150 mm analytical column filled with Zorbax 300SB-C18, 5 μm (Agilent Technologies) was used for peptide separation.

The identification of peptides was based on data collected in auto MS/MS 2 GHz mode using the following settings; fragmentor: 175 V, skimmer: 65 V, OCT 1 RF Vpp: 750 V, precursor ion selection: medium (4 m/z), mass range: 200–2500 m/z, acquisition rate for MS: 2 spectra/sec, for MS/MS 3 spectra/sec; MS/MS range: 50–3000 m/z; ramped collision energy: slope 3.8, offset: 0, precursor setting: maximum 3 precursors/cycle; absolute threshold for peak selection was 1000; relative threshold was 0.01% of the most intense peak, active exclusion enabled after 1 selection, release of active exclusion after 0.6 min, precursors were sorted by abundance only. The MS/MS files were stored in centroid and profile mode. MS1 absolute threshold 50 and MS2 absolute threshold 35 were applied to account for detector noise. Static exclusion range 200–350 m/z for precursor selection was applied. Gas temperature (nitrogen) was 325 °C and gas flow was 5 l/min. The quantification of peptides was based on data collected in MS mode using the same settings except of the mass range 20–3000 m/z; acquisition rate 1 spectra/sec. In both cases lock masses 1221.990 m/z and 299.294 (Agilent) were used to recalibrate spectra during the acquisition. The area of the manually extracted ion chromatograms (0.1 m/z tolerance) of the selected peptides was used for quantification.

### Database search

Tandem mass spectra were extracted, charge state deconvoluted and deisotoped by the MassHunter Qualitative Analysis software version B.05.00 (Agilent) and saved as. mgf files. All MS/MS data were analyzed using Phenyx (GeneBio, Geneva, Switzerland); version CYCLONE (2010.12.01.1)). The fragment ion mass tolerance of 0.30 Da and a parent ion tolerance of 400 ppm were selected for a database search. The oxidation of methionine (+15.99), light (+42.01) and heavy (+45.03) acetylation of lysine (K), heavy acetylation with light methylation (+59.045) of K, methylation (+14.01), dimethylation (+28.03), trimethylation (+42.04) of K, methylation of arginine (R) (+14.01), dimethylation of arginine (R) (+28.03) N-terminal acetylation (+42.01), deamidation (+0.98) of asparagine (N) and glutamine (Q) were specified in Phenyx as variable modifications.

### Nuclear and cytosolic fractionation

RAW264.7 macrophages were subjected to 1 μM MS-275 and 0.41 μM SAHA for 20 h, stimulated with 10 ng/mL LPS and 10 ng/mL IFNγ for 4 h and subsequently lysed. Nuclear and cytosol fractions of RAW264.7 macrophages were prepared using the NE-PER^®^ Nuclear and Cytoplasmic Extraction kit (Thermo Scientific) according to the protocol of the manufacturer.

### Confocal laser scanning microscopy (CLSM)

The effect of the MS-275 and SAHA on the nuclear translocation of NF-κB p65 was evaluated by immunofluorescence. 20,000 cells/cm^2^ cells were seeded in Lab-Tek chambers (#177445; Nunc, Rochester, NY, USA) and subjected to 1 μM MS-275 and 0.41 μM SAHA for 20 h. Cells were washed twice with ice-cold DPBS and fixed with 4% formaldehyde at room temperature for 20 minutes. Subsequently, cells were permeabilized with 0.25% Triton X‐100 in DPBS and blocked with DPBS/5% BSA for 30–60 min at room temperature (to minimize non-specific binding). NF-κB p65 was detected with rabbit monoclonal NF-κB p65 antibody (2 h incubation at 4 °C, 1:250; #8242, Cell Signaling), followed by 1 h incubation with goat anti-rabbit AlexaFluor_488_ (10 μg/mL; A-11008, Molecular Probes, Leiden, The Netherlands). All antibody incubation steps were carried out in 5% BSA/DPBS and between incubation steps slides were washed with DPBS/0.5% BSA/0.05% Tween 20. For negative control, samples were processed omitting the primary antibody. At the end of the incubation steps, nuclei were stained with Hoechst 33342 (Molecular Probes) and mounted using Aqua Poly/Mount medium (Polysciences, Warrington, PA, USA), air dried for 24 h, and stored in the dark at 4 °C. Fluorescence was examined using a confocal laser scanning microscope (CLSM) equipped with true confocal scanner (TCS; SP8 Leica, Heidelberg, Germany), using a 63x oil immersion lens. Sequential scans were obtained to avoid bleed through. AlexaFluor_488_ was excited using the 488 nm blue laser line, AlexaFluor_546_ was excited using the 552 nm green laser line and Hoechst 33342 using the 405 nm UV laser line. All images were recorded in the linear range, avoiding local saturation, at an image resolution of 2048 × 2048 pixels and with a pinhole size of 1 Airy unit. Presented images show a single z-scan. Images were further processed using ImageJ 1.48d.

### Chromatin Immunoprecipitation (ChIP)

RAW264.7 macrophages were plated in 175 cm^2^ flasks and subjected to 1 μM MS-275 or vehicle for 20 h from which the last 4 h supplemented with 10 ng/mL LPS/IFNγ. RAWs were cross-linked for 20 min at RT with 1.5 mM EGS in PBS followed by 15 min with 1% formaldehyde (#28906, methanol-free; Thermo Scientific). Cross-linking was quenched with 200 mM glycine in PBS for 5 min and subsequently cells were washed 3 times with ice-cold PBS. Per ChIP, 25–30 × 10^6^ cells were lysed with scrape buffer (PBS supplemented with 3 μL Pierce^TM^ Protease Inhibitors (P.I.); #88266, Thermo Scientific) and centrifuged at 500 g for 5 min at 4 °C. To isolate the nuclei, the pellets were incubated in 1 mL LB1 buffer (50 mM Hepes-KOH, pH 7.5, 140 mM NaCl, 1 mM EDTA, 10% glycerol, 0.5% IGEPAL CA-630 and 0.25% Triton-X 100, supplemented with 3 μL/mL P.I.)/25 × 10^6^ cells and rotated for 20 minutes at 4 °C to ensure efficient lysis. After centrifugation at 2000 g for 5 minutes at 4 °C, nuclei were washed in LB2 buffer (10 mM Tris-HCl, pH 8.0, 200 mM NaCl, 1 mM EDTA and 0.5 mM EGTA, supplemented with 3 μL/mL P.I.), for 5 minutes at 4 °C with rotation and afterwards centrifuged at 2000 g for 5 minutes at 4 °C. Nuclei were lysed in LB3 buffer (10 mM Tris-HCl, pH 8.0, 100 mM NaCl, 1 mM EDTA, 0.5 mM EGTA, 0.1% Na-deoxycholate and 0.5% lauroylsarcosine, supplemented with 3 μL/mL P.I.) and sonicated with a Bioruptor^®^ Plus device (Diagenode Inc., USA) to fragment the chromatin to a mean size of 300 bp. 25 μg of pre-cleared sonicated chromatin was incubated overnight on a rotating platform at 4 °C with Protein-A Dynabeads^®^ (#10001D, Thermo Scientific) prebound with 1–2 μg of control IgG (#3900, Cell Signaling) or NF-κB p65 (#8242, Cell Signaling) antibodies. Afterwards, the immune complexes were washed seven times with RIPA buffer (50 mM Hepes-KOH, pH 7.6, 1 mM EDTA, 0.7% Na-deoxycholate, 1% IGEPAL CA-630, 0.5 M LiCl) and two times with TE buffer (10 mM Tris-HCl and 1 mM EDTA, pH 8.0). ChIP DNA fragments were eluted and decross-linked by incubation with elution buffer (1% SDS, 0.1 M NaHCO_3_) overnight at 65 °C. Next the DNA fragments were incubated with 1 mg/mL RNase (#11119915001, Roche) for 1 h at 37 °C and subsequently incubated with 20 mg/mL Proteinase K (#03115887001, Roche) for 2 h at 55 °C to reduce residual RNA and protein. Finally, ChIP DNA fragments were isolated using the Wizard^®^ SV Gel and PCR Clean-Up System (Promega) according to the manufacturer’s protocol and quantified by RT-qPCR using an ABI Prism 7900HT Sequence Detection System (Applied Biosystems) and a SensiMix^TM^ SYBR^®^ Hi-ROX Kit (#QT605-05, Bioline, Alphen aan den Rijn, The Netherlands). The following *IL-10* primers were used: *−*48 bp transcription start site, 5′-TCAAAAATTGCATGGTTTAGAAGA-3′ (FW), 5′-TGTTCTTGGTCCCCCTTTTA-3′ (RV); *−*220 bp transcription start site, 5′-AGCTGTCTGCCTCAGGAAAT-3′ (FW), 5′-TGGTCGGAATGAACTTCTGC-3′ (RV). qChIP data were calculated as percent of input and plotted as fold enrichment.

### Mouse model of cigarette smoke exposure

Male mice were exposed (whole body) twice a day for 4 consecutive days to cigarette smoke from research cigarettes (Kentucky 3R4F), as described previously^56^. In brief, on day 1, mice were exposed to the mainstream smoke of one cigarette in the morning and three cigarettes in the afternoon, and on day 2–4, five cigarettes in the morning and five cigarettes in the afternoon (Fig. 6A) (1 cigarette corresponds to 5 minutes of exposure followed by 30 seconds of air exposure and subsequently 2 minutes of recovery). 30 minutes prior to each cigarette smoke exposure mice were subjected to 10 μM MS-275 by nebulization (using a PARI BOY SX pump type 085 (Pari GmbH, Starnberg, Germany) in PBS 1% DMSO for 15 minutes. Vehicle treated mice were exposed to PBS 1% DMSO (no MS-275) and cigarette smoke. Control mice were handled in the same manner, however, exposed to fresh air only. 16 h after the last cigarette smoke/air exposure mice were anesthetized by subcutaneous injection of ketamine (40 mg/kg) and dexdomitor (0.5 mg/kg). Subsequently, BAL fluid was obtained and lungs were snap frozen on liquid nitrogen and stored at −80 °C. Animal experiments were performed according to national guidelines and upon approval of the experimental procedures by the local Animal Care and Use committee of Groningen University, DEC number 6962B. Mice were randomly assigned to the experiments. The number of mice available for analysis per group is shown in the figures.

### Analysis of BAL fluid

After anesthetizing the mice, lungs were gently lavaged through a tracheal cannula with 1 mL PBS containing 5% BSA supplemented with protease inhibitors (# 11836170001, Roche, 1 tablet per 10 mL) and another 4 times with 1 mL PBS. Bronchoalveolar lavage (BAL) fluid was centrifuged at 290 g for 10 minutes at 4 °C and the supernatants of the first fractions were stored at −80 °C and analyzed for KC (IL-8) content by ELISA (#DY453-05, R&D Systems, Minneapolis, MN, USA). For each animal individually, BAL cells of the 5 different fractions were combined, resuspended in 500 μL PBS, and total cell numbers were determined. For cytological determination, cytospin preparation were stained with May-Grünwald and Giemsa (both Sigma) and a differential cell count was performed by counting ≥400 cells in duplicate in a double blinded fashion.

### Immunohistochemical staining and quantification of CD68^+^ and CD68^+^/IL-10^+^ macrophages in lung tissue of cigarette smoke-induced neutrophilic airway inflammation

To determine the number of CD68^+^ and CD68^+^/IL-10^+^ macrophages in lung tissue, 4 μm-thick cryosections (n = 4–5) were stained with specific antibodies. First, cryosections were fixed for 10 minutes in 100% acetone and subsequently blocked for 30 minutes with 5% ELK and 1% BSA in PBS. Next, sections were incubated overnight at 4 °C with 10 μg/mL anti-human IL-10 rabbit IgG (#HP9016, Hycult Biotech, Uden, The Netherlands). Endogenous peroxidase activity was blocked with 0.3% H_2_O_2_ and endogenous biotin was blocked using a Biotin Blocking System (#X0590, Dako). Subsequently, sections were incubated with 4 μg/mL rat anti-mouse CD68 (#MCA1957GA, Abd Serotec^®^, Bio-Rad) for 1 h, followed by incubation with 7.6 μg/mL polyclonal goat anti-rabbit immunoglobulins/biotinylated (#E0432, Dako) for 30 minutes and 1:50 goat anti-rat IgG/HRP (#3050-05, Southern Biotech, Birmingham, USA) for 30 minutes. Immunoreactivity was visualized using a Vectastain ABC Kit (#PK-4000, Vector Laboratories, Burlingame, USA), an BCIP/NBT Alkaline Phosphatase Substrate Kit (#SK-5400, Vector Laboratories; 5-bromo-4-chloro-3-indolyl phosphate/nitro blue tetrazolium) and an ImmPACT^TM^ NovaRED^TM^ Peroxidase Substrate Kit (#SK-4805, Vector Laboratories), according to manufacturer’s protocols. Finally, sections were dehydrated through 95% and 100% alcohol and mounted with VectaMount^TM^ Permanent Mounting Medium (#H-5000, Vector Laboratories). As negative control, sections were processed omitting the primary antibody. CD68-positive cells and double-positive cells were counted manually and corrected for the total area of lung tissue section as measured by morphometric analysis using Aperio ImageScope viewing software 11.2.0.780 (Aperio, Vista, USA).

### HDAC activity in nuclear lung extracts

To determine the HDAC activity in lung tissue of cigarette smoke-exposed mice treated with HDAC1-3 inhibitor MS-275, nuclear fractions were prepared. First, 25 mg lung tissue was washed with 1 mL ice-cold PBS and centrifuged at 500 g for 5 min at 4 °C. Subsequently, the supernatant was removed and nuclear fractions were prepared using the NE-PER^®^ Nuclear and Cytoplasmic Extraction kit (Thermo Scientific) according to the protocol of the manufacturer.

The HDAC activity in the resulting nuclear lung extracts was determined using an assay described before^34^. Shortly, nuclear extracts were serial diluted in 40 μL of HDAC buffer (25 mM Tris-HCl, 137 mM NaCl, 2.7 mM KCl and 1 mM MgCl_2_). Negative control contained 40 μL heat inactivated nuclear extracts; 100 °C for 10 min. Subsequently, 50 μl of Boc-Lys(Ac)-AMC (100 μM) and 10 μl of HDAC buffer was added to all wells (final volume 100 μL). The plates were gently shaken for 20 sec and incubated at room temperature for 1 h. Next, 50 μl of HDAC stop solution (300 μM SAHA supplemented with 6 mg/mL trypsin) was added to all wells and the plate was incubated at 37°C for 20 min. The fluorescence was measured at *λ*_em_ = 460 nm and *λ*_ex_ = 390 nm. Arbitrary fluorescence units (AFU), corresponding to HDAC activity, were plotted versus mg protein for all samples.

### Statistical analysis

The data are presented as mean ± SD unless stated otherwise. Data are derived from three or more independent experiments (unless otherwise indicated) and were analyzed with the Prism 5.0 statistical program from GraphPad Software. Statistical analysis of the data was performed by a two-tailed unpaired Student’s *t*-test, assuming equal variances to compare two replicate groups. Analysis of differences between multiple replicate groups was analyzed with one-way ANOVA followed by Bonferroni or Tukey *post hoc* analysis. *p* values ≤0.05 were considered to be significant.

## Additional Information

**How to cite this article**: Leus, N. G. J. *et al*. HDAC1-3 inhibitor MS-275 enhances *IL10* expression in RAW264.7 macrophages and reduces cigarette smoke-induced airway inflammation in mice. *Sci. Rep.* **7**, 45047; doi: 10.1038/srep45047 (2017).

**Publisher's note:** Springer Nature remains neutral with regard to jurisdictional claims in published maps and institutional affiliations.

## Supplementary Material

Supplementary Information

## Figures and Tables

**Table 1:** IC_50_ values of HDAC1-3-selective inhibitor MS-275 and pan-HDAC inhibitor SAHA for recombinant human HDAC1-3, HDAC 6 and HDAC 8.

	HDAC 1	HDAC 2	HDAC 3	HDAC 6	HDAC 8
MS-275	0.19 ± 0.04	0.41 ± 0.09	0.95 ± 0.19	≥100	76.5 ± 10.1
SAHA^a^	0.07 ± 0.01	0.16 ± 0.05	0.05 ± 0.02	0.06 ± 0.01	1.52 ± 0.46

Data are presented as mean values (in μM) of 3 independent measurements ± SD. ND = not determined. ^a^IC_50_ values for SAHA are taken from Szymanski *et al*.^34^.
