# Supplementary material for: HDAC1-3 inhibitor MS-275 enhances IL10 expression in RAW264.7 macrophages and reduces cigarette smoke-induced airway inflammation in mice
Source: Sci Rep. 2017 Mar 27;7:45047. doi: 10.1038/srep45047 (PMC5366870; doi:10.1038/srep45047)
Supplement: Supplementary Information [file srep45047-s1.pdf]

Supporting Information

HDAC1-3 inhibitor MS-275 enhances *IL10* expression in in RAW264.7 macrophages and reduces cigarette smoke-induced airway inflammation in mice

Niek G.J. Leus[a*], Thea van den Bosch[a*], Petra E. van der Wouden[a*], Kim Krist[a*], Maria E. Ourailidou[a], Nikolaos Eleftheriadis[a], Loes E.M. Kistemaker[b], Sophie Bos[b], Rutger A.F. Gjaltema[c], Solomon A. Mekonnen[a], Rainer Bischoff[d], Reinoud Gosens[b], Hidde J. Haisma[a] and Frank J. Dekker[a#]

[*] These authors contributed equally to this work

Table S1. Concentration of HDAC inhibitors used for MTS assay

	MS-275	SAHA
RAW 264.7	1 µM	0,41 µM

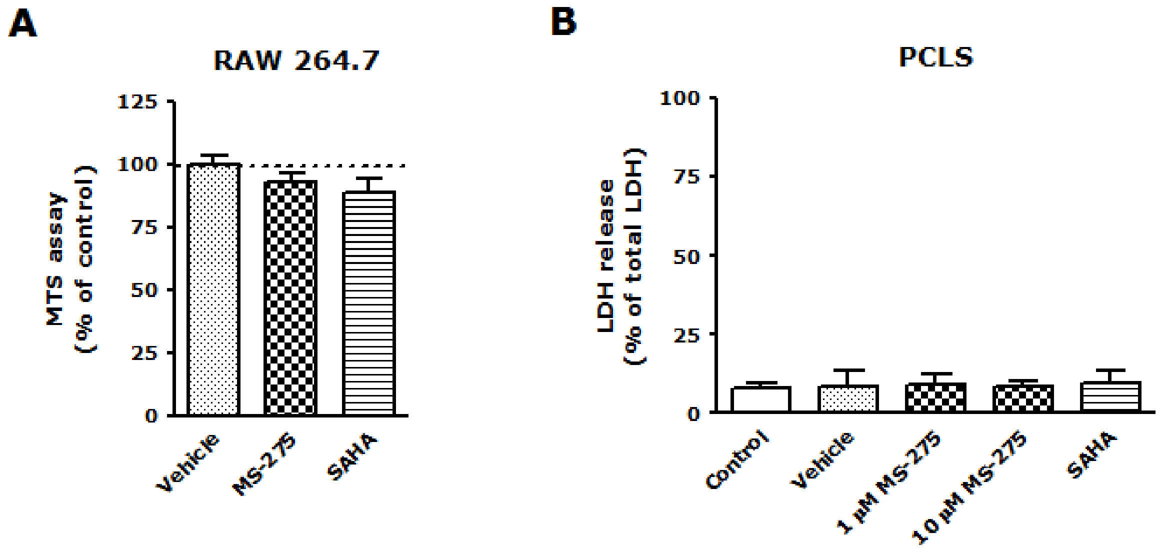

Figure S1. Cell and tissue viability upon incubation with HDAC 1-3-selective inhibitor MS-275 and pan-HDAC inhibitor SAHA. RAW 264.7 macrophages were incubated with HDACi at indicated concentrations (see Table S1) for 20 h. After the incubation, viability of cells was investigated using CellTiter 96 AQ_{ueous} One Solution reagent as described in *Materials and Methods*. The viability of cells without addition of vehicle or HDACi was considered to be 100%. Data are presented as mean values ± SD of 3 independent experiments. In parallel, the lactate dehydrogenase (LDH) release **(B)** from mouse precision-cut lung slices (PCLS) were analyzed. Total LDH content of the slices was determined by lysis with 1% Triton X-100. LDH release was plotted relative to total LDH and presented as mean values ± SD of 4 independent experiments.

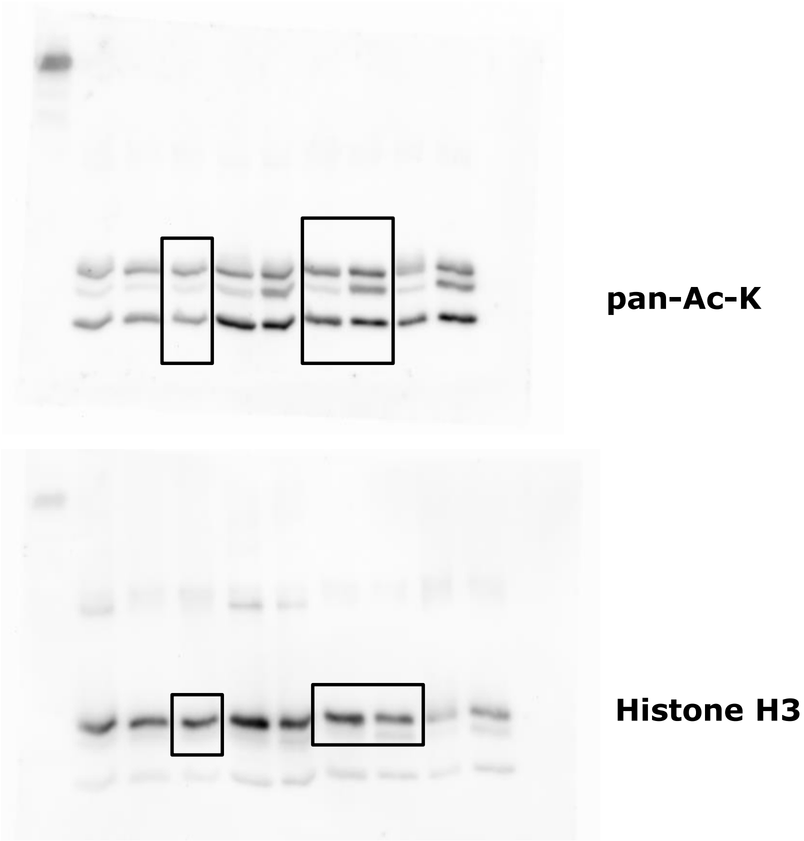

Figure S2. The uncropped blots of Figure 1C. The black boxes indicate which bands are shown in the main paper.

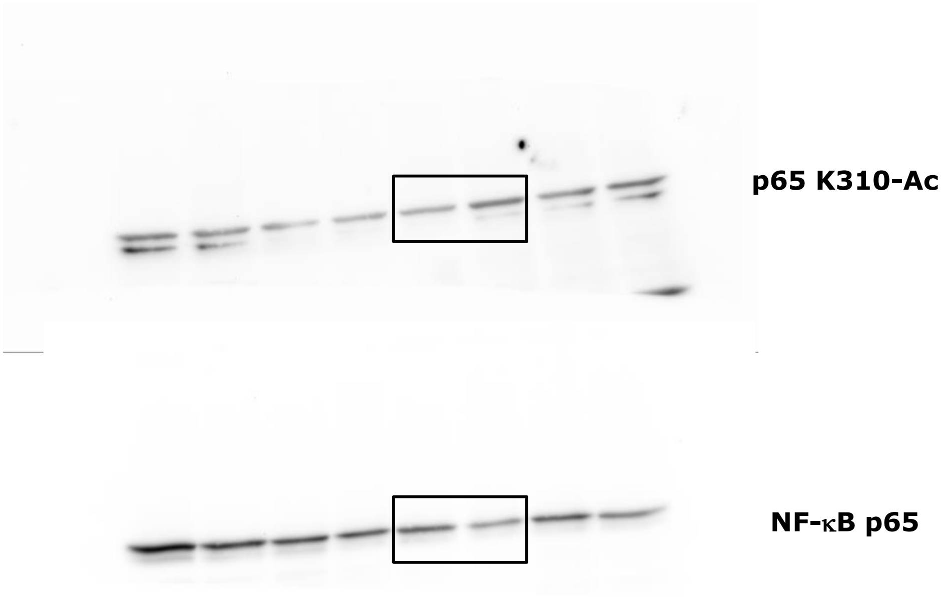

Figure S3. The uncropped blots of Figure 3B. The black boxes indicate which bands are shown in the main paper.

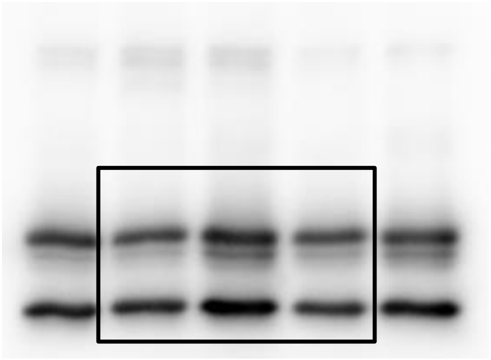

Figure S4. The uncropped blot of Figure 3D. The black box indicates which bands are shown in the main paper.

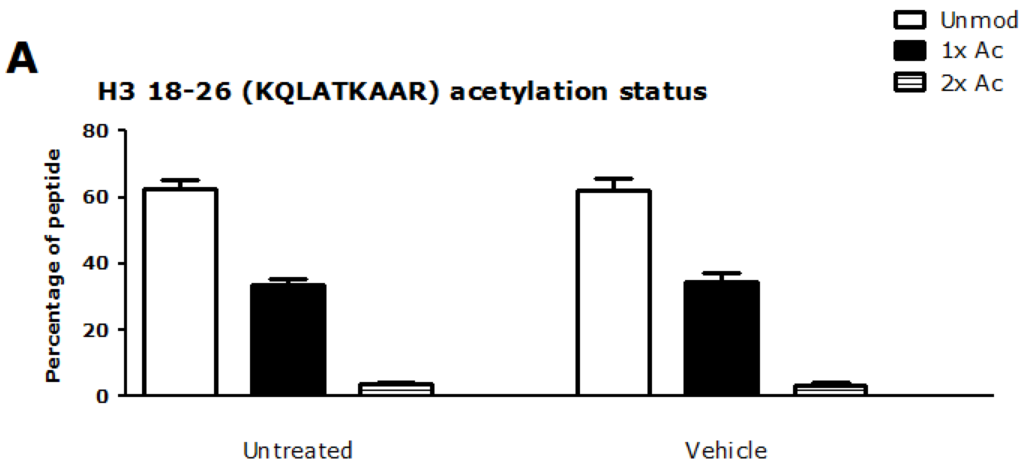

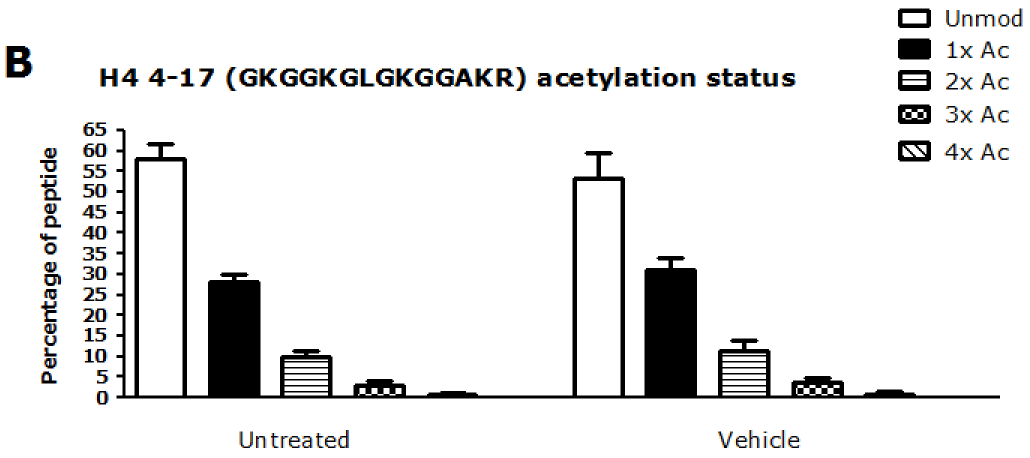

Figure S5. No effect of the vehicle treatment on histone acetylation in RAW264.7 macrophages. RAW264.7 histones were resolved by SDS-PAGE and histones H3 and H4

were excised from the gel and subjected to LC-MS/MS analysis. There was no effect on (A) the peptide of histone H3 (res. 18-26: KQLATKAAR), nor on (B) the peptide from histone H4 (res. 4-17: GKGGKGLGKGGAKR). Data are presented as mean ± SD of 4-5 independent experiments.

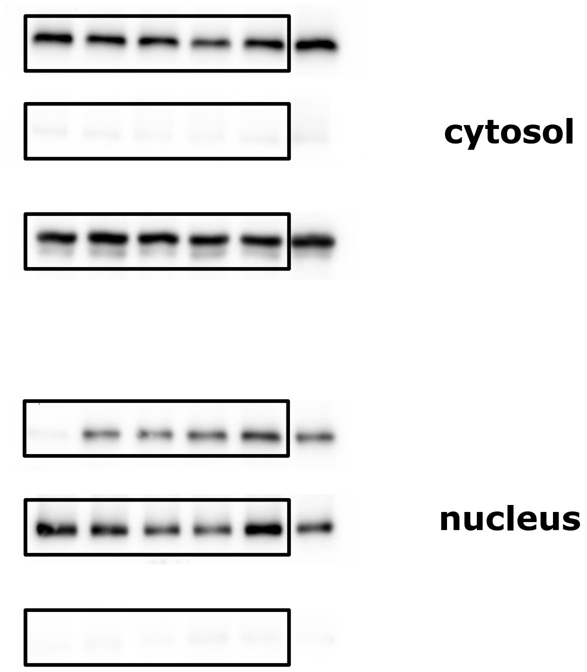

Figure S6. The uncropped blots of Figure 4B. The black boxes indicate which bands are shown in the main paper.

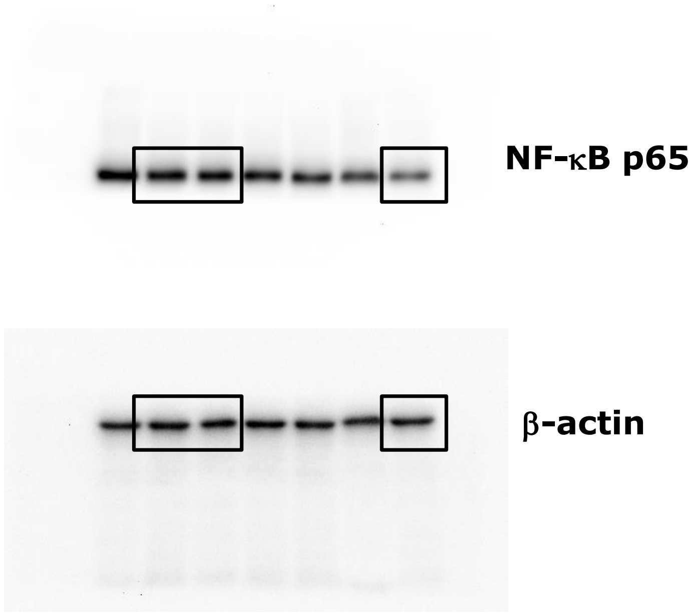

Figure S7. The uncropped blots of Figure 4D. The black boxes indicate which bands are shown in the main paper.

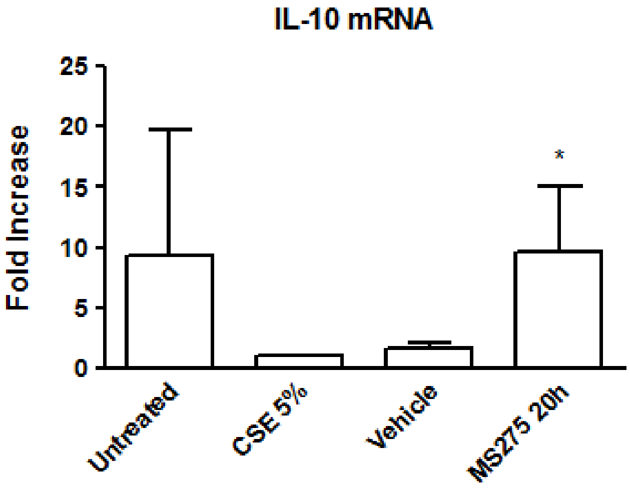

Figure S8. RAW264.7 macrophages exposed to cigarette smoke extract (CSE) show increased IL-10 expression upon MS-275 treatment. Cells were treated with MS-275 or vehicle for 20 hrs, or were left untreated, and where appropriate were exposed to CSE the last 4 hrs of the experiment. Gene expression was studied by RT-qPCR and expressed as fold

change compared to control (LPS/IFNγ-treated) group. Data are presented as mean ± SD of 3-4 independent experiments. * $p < 0.05$ compared to vehicle (LPS/IFNγ and inhibitor solvent-treated).

Table S2. Effect of HDAC1-3 inhibitor MS-275 on liver and kidney function parameters

Treatment	ALAT (U/l)	ASAT (U/l)	ALP (U/l)	LDH (U/l)	Creatinine (µmol/l)	Urea (mmol/l)	eGFR (ml/min)
**Air exposure**	**28.0 ± 6.1**	**142 ± 84**	**58.3 ± 8.1**	**323 ± 105**	**10.0 ± 2.7**	**9.9 ± 1.8**	**261 ± 24**
**Cigarette smoke exposure vehicle**	**34.0 ± 12.9**	**156 ± 119**	**54.8 ± 12.8**	**346 ± 117**	**10.2 ± 2.3**	**9.3 ± 1.1**	**257 ± 23**
**Cigarette smoke exposure 10 µM MS-275**	**31.4 ± 9.8**	**232 ± 170**	**55.4 ± 11.0**	**376 ± 100**	**11.6 ± 3.9**	**9.4 ± 0.9**	**251 ± 33**

Blood samples were taken from air exposed and cigarette smoke exposed mice subjected to either vehicle or 10 µM MS-275 and analyzed for blood levels of the enzymes alanine aminotransferase (ALAT), alanine aminotransaminase (ASAT), alkaline phosphatase (ALP), lactate dehydrogenase (LDH), creatinine, urea, and glomerular filtration rate (eGFR). Values are presented as mean values ± SD; n=4-5 mice per group. No loss in liver function was observed, as levels of the liver enzymes ALAT, ASAT, ALP and LDH were not increased compared to air-exposed mice. In addition, no differences in plasma levels of creatinine and urea were observed. Moreover, eGFR remained unaffected in mice subjected to cigarette smoke and MS-275 or vehicle, indicating maintenance of regular kidney function.

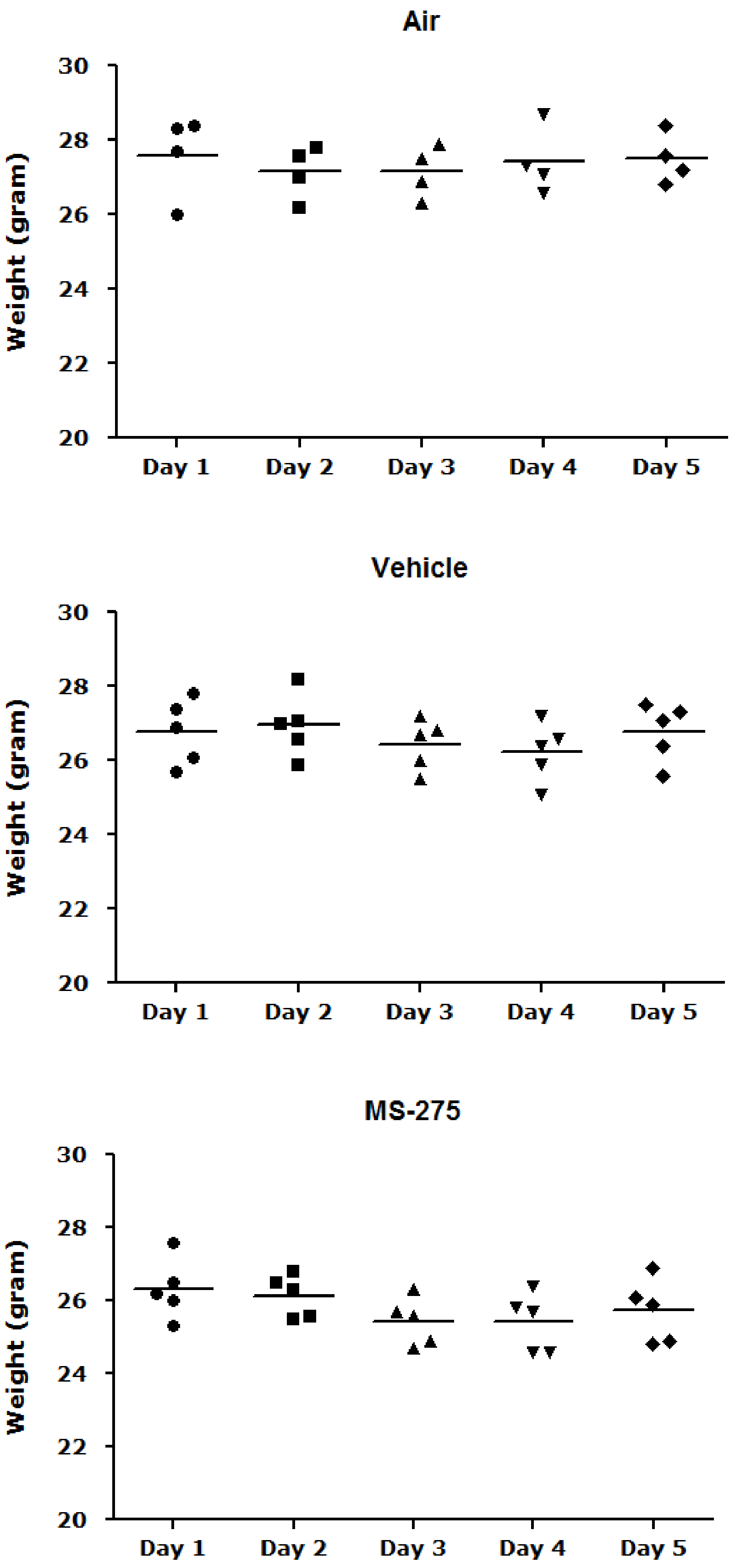
Air
Weight (gram)
30
28
26
24
22
20
Day 1
Day 2
Day 3
Day 4
Day 5
Vehicle
Weight (gram)
30
28
26
24
22
20
Day 1
Day 2
Day 3
Day 4
Day 5
MS-275
Weight (gram)
30
28
26
24
22
20
Day 1
Day 2
Day 3
Day 4
Day 5

Figure S9. No changes in the weight of the animals upon MS-275 treatment. Throughout the experiment no changes in weight were observed between the air, vehicle or MS-275 treated mice.

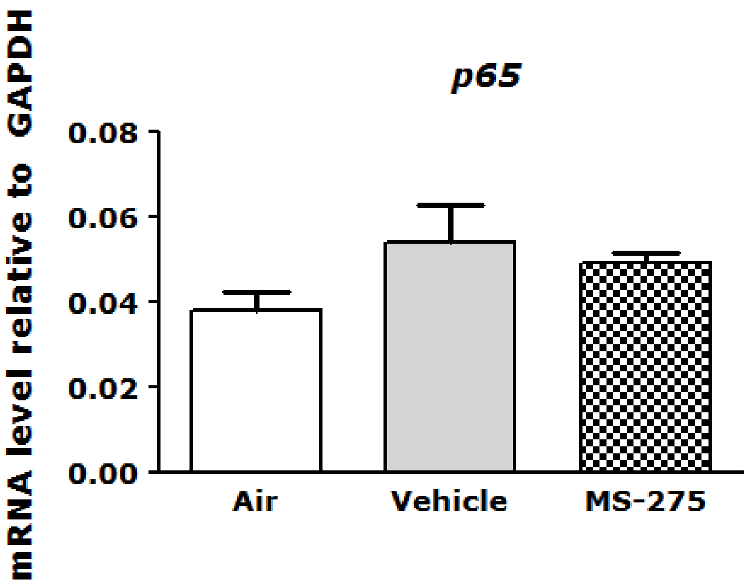

Figure S10. No effects were observed on p65 mRNA levels in lung tissue homogenates from the mice. Gene expression in lung homogenates was studied by RT-qPCR and expressed relative to GAPDH.

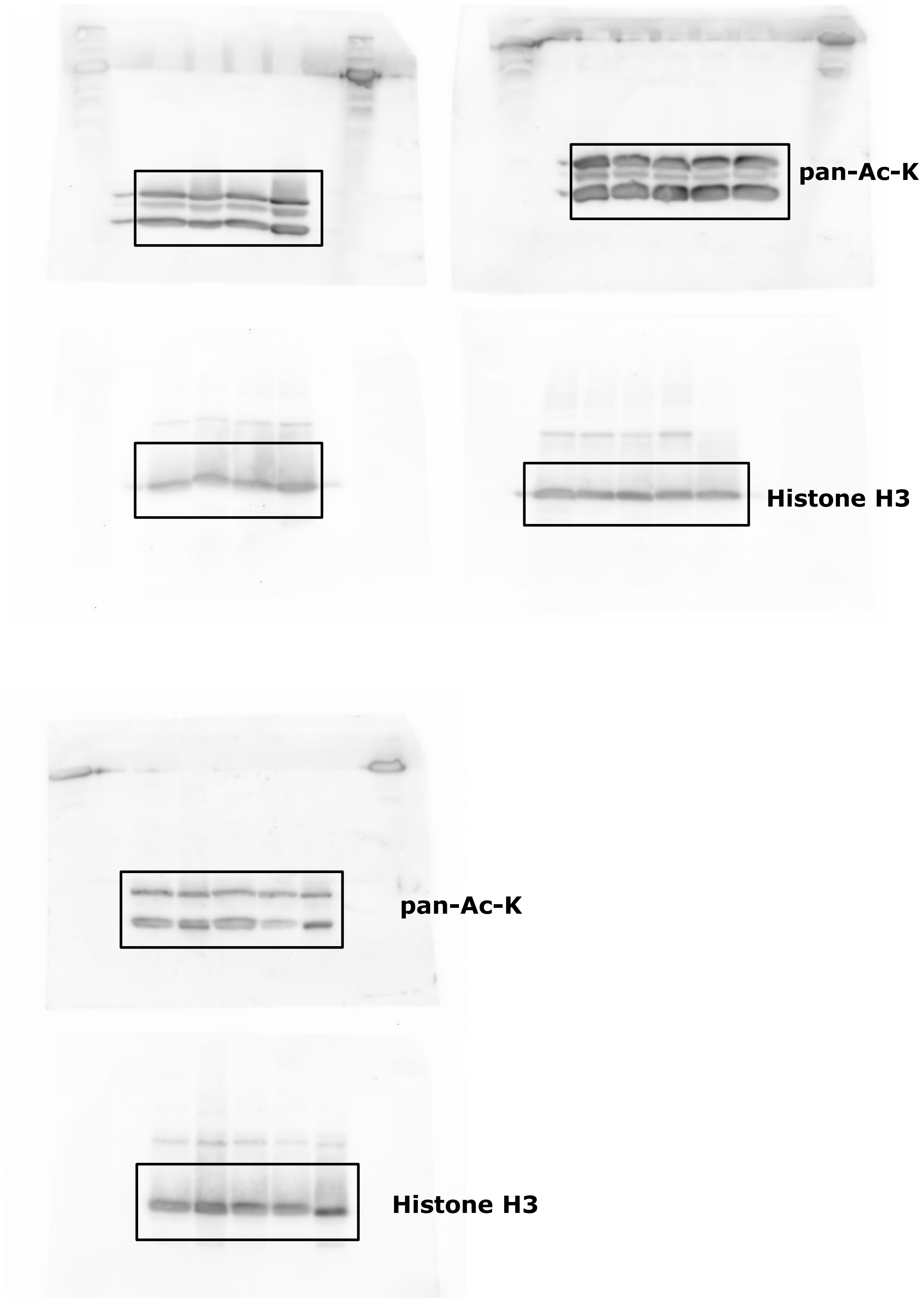

Figure S11. The uncropped blots for Figure 7B. The black boxes indicate which bands are shown in the main paper.
